# CLCAs - A Family of Metalloproteases of Intriguing Phylogenetic Distribution and with Cases of Substituted Catalytic Sites

**DOI:** 10.1371/journal.pone.0062272

**Published:** 2013-05-09

**Authors:** Anna Lenart, Małgorzata Dudkiewicz, Marcin Grynberg, Krzysztof Pawłowski

**Affiliations:** 1 Department of Cellular and Molecular Neurobiology, Nencki Institute of Experimental Biology, Polish Academy of Sciences, Warsaw, Poland; 2 Faculty of Agriculture and Biology, Warsaw University of Life Sciences, Warsaw, Poland; 3 Department of Genetics, Institute of Biochemistry and Biophysics, Polish Academy of Sciences,Warsaw, Poland; Macquarie University, Australia

## Abstract

The zinc-dependent metalloproteases with His-Glu-x-x-His (HExxH) active site motif, zincins, are a broad group of proteins involved in many metabolic and regulatory functions, and found in all forms of life. Human genome contains more than 100 genes encoding proteins with known zincin-like domains. A survey of all proteins containing the HExxH motif shows that approximately 52% of HExxH occurrences fall within known protein structural domains (as defined in the Pfam database). Domain families with majority of members possessing a conserved HExxH motif include, not surprisingly, many known and putative metalloproteases. Furthermore, several HExxH-containing protein domains thus identified can be confidently predicted to be putative peptidases of zincin fold. Thus, we predict zincin-like fold for eight uncharacterised Pfam families. Besides the domains with the HExxH motif strictly conserved, and those with sporadic occurrences, intermediate families are identified that contain some members with a conserved HExxH motif, but also many homologues with substitutions at the conserved positions. Such substitutions can be evolutionarily conserved and non-random, yet functional roles of these inactive zincins are not known. The CLCAs are a novel zincin-like protease family with many cases of substituted active sites. We show that this allegedly metazoan family has a number of bacterial and archaeal members. An extremely patchy phylogenetic distribution of CLCAs in prokaryotes and their conserved protein domain composition strongly suggests an evolutionary scenario of horizontal gene transfer (HGT) from multicellular eukaryotes to bacteria, providing an example of eukaryote-derived xenologues in bacterial genomes. Additionally, in a protein family identified here as closely homologous to CLCA, the CLCA_X (CLCA-like) family, a number of proteins is found in phages and plasmids, supporting the HGT scenario.

## Introduction

The protein sequence space, recently becoming sampled more and more densely thanks to genomic and metagenomic sequencing projects, has undoubtedly ‘granular’ features, and can be classified using various algorithms and classification systems [1], [2]. Yet, it has also features of continuity, with very distant sequence similarities discovered between hitherto unrelated protein families, and local structural similarities found between members of different folds [3], [4]. The protein sequence/structure space, arguably, is not fully sampled during evolution. Further, the still incomplete charting of the protein universe is expected to be biased by technology and prevailing research trends [5], [6]. Even for the charted regions of the sequence space, many evolutionarily-justified similarity relationships are not obvious and often are found only after solving three-dimensional structures [7] or applying sophisticated bioinformatics approaches [8]–[10].

Here, we focus on a broad clan of metalloproteases that are a good example of protein class with such a dual granular and continuous features. The proteases, originally noted for their involvement in digestive processes, are now acknowledged for many crucial regulatory roles in cellular signalling in diverse biological processes, on cellular, tissue and organism scale, e.g. in cell proliferation and differentiation, inflammation, tissue remodelling, neurogenesis, angiogenesis, apoptosis, wound healing, blood coagulation [11]–[14]. Not surprisingly, proteases constitute an important class of drug targets [15]–[17]. Among the generic class of proteases, distinct clans have been identified using the catalytic mechanism and three-dimensional fold as the classifier [18], [19]. The zinc ion-dependent zincin-like metalloproteases grouped in the MA clan in the MEROPS database include 37 families [20], while in the Pfam database there are 52 families of the Peptidase_MA clan, including also putative metalloproteases [21], [22]. The proteins containing the zincin-like domains often feature complex domain composition reflecting their biological functions [23].

Here, we explore the realm of all proteins identified by the simple HExxH active site motif common to most MA clan member families and show topology features of the sequence similarity network of the clan families. Also, we show that the motif can be used as a prefilter for discovery of novel metalloproteases.

CLCAs are a protein family implicated in several pathologies in humans, including asthma, chronic obstructive pulmonary disease (COPD) and cancer [24], [25]. Originally, they were believed to be calcium-activated chloride channels [26], [27]. Despite their characterisation as putative metalloproteases several years ago [28], they attracted moderate interest. The current view is that they are involved in regulation of calcium-activated chloride currents [29]. Several members of the CLCA family have been characterised beyond any doubt as secreted zinc-dependent metalloproteases [30], [31] that perform self-cleavage at a conserved site. Vertebrates possess several closely homologous CLCA genes (usually 3–6), the functional relationships between them are not fully elucidated. It is not known whether CLCAs possess other physiological substrates except themselves, whether they are cleaved by other proteases except themselves, and whether different CLCA proteins cleave each other [32], [33]. It is now believed that activation of ion channels by CLCA proteins occurs via a direct protein-protein interaction between an ion channel molecule and the N-terminal fragment of a CLCA protein, an interaction possible only after CLCA self-cleavage [31].

Recently, cases of patchy phylogenetic distribution of homologues of human genes in prokaryotes have attracted some attention, [9], [10], [23]. Such distribution has been interpreted as potential sign of horizontal gene transfer (HGT) [34]–[37].

In this article, first, we survey the HExxH proteins and identify domain families with majority of members containing the motif. Second, we analyse the substituted HExxH motifs in families where they are conserved in most members. Third, we provide support for the hypothesis of eukaryote to prokaryote horizontal gene transfer in CLCA proteins. Lastly, we present the prokaryote-specific CLCA-like domain family (CLCA_X) and present an overall representation of sequence similarity topology of the zincin-like clan.

## Results and Discussion

### Survey of HExxH motif-containing proteins

The ubiquitous HExxH zinc-binding motif is a hallmark of zinc-dependent metalloproteases [38]–[41]. We surveyed the Trembl database and found 151223 occurrences of the motif compared to 80000 expected by chance (significant, almost twofold over-representation, p-value of the binomial text <<10^−10^, see Methods). Then, we checked whether the occurrences of this motif were within the known Pfam protein domains (Pfam database version 24.0), or outside those.

After removal of redundancy in the hit sequence set at 90% sequence identity, the occurrence of the HExxH motif within Pfam domains was 47794 (versus 41946 expected) which makes up 52% of the occurrences, while the occurrence outside Pfam domains was 43395 (versus 49242 expected). Thus, since approx. 46% of the total length of Trembl proteins lie within the Pfam domains, within these domains the HExxH motif is found significantly more often (p-value of the binomial text <<10^−10^, see Methods) than expected by chance, while outside of the domains it is found significantly less often than expected.

The regions of protein sequence databases unassigned to known protein domains (e.g. Pfam) can be unassigned for two reasons: first, they may constitute novel, yet undescribed domains, second, they may belong to special regions (e.g. transmembrane segments, low-complexity regions, disordered regions, unique variable regions and so forth). Hence, it can be expected that some HExxH motifs found here outside Pfam domains actually do occur in yet undiscovered protein domains, possibly in novel protease domains. Search for novel metalloprotease domains is out of the scope of this article, however the existence of yet undescribed zincin domains can be argued for by the rapid increase in the numbers of zincin domains described in domain databases (for example, in the Pfam database, the Peptidase_MA clan grew from 36 families in release 24, 2009, to 52 families in release 26, 2011 [42]. Also, recently, some novel zincin families have been characterised [23], [30]. Examples of zincin-like metalloprotease domains, suspected but not described formally yet, include the family of the ddrB protein of *E. coli* bacteriophage P1 [43] and the CLCA_X family mentioned herein.

Since the HExxH motif is found in protein sequences twice as often as expected by chance, even if the HExxH metalloproteases had not been known, one would have expected some functional relevance of the motif. Yet, obviously, some of the occurrences have to be due to chance. One way of separating the functional HExxH motifs from the random ones is identifying those motifs that are conserved by evolution. To this extent, we sought protein domain families, for which significant fraction of family members had the motif present in a conserved position.

**Figure 1:**
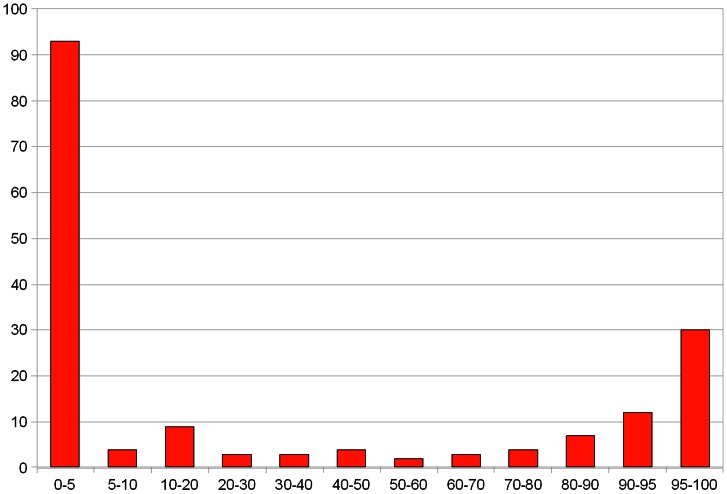
Histogram. Pfam protein domains binned by percentage of family members that possess the HExxH motif. Shown are only the domains with more than 50 occurrences of the motif included plus all Peptidase domains with at least one occurrence.

**Figure 2:**
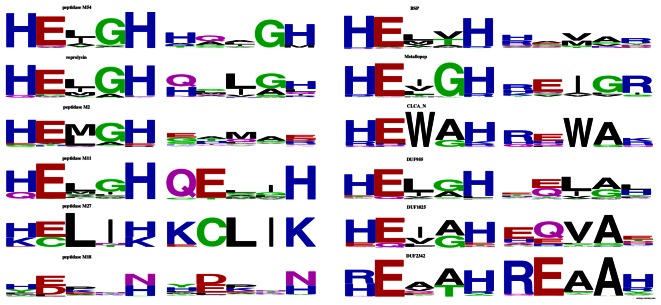
Sequence logos of substituted and conserved active site motifs in selected zincin-like families.

The Pfam domains are clearly split into those that have few (sporadic, random) occurrences of HExxH and those that possess the motif, as a rule, in a conserved position (see Fig. 1). Indeed, all the domains with majority of members having a HExxH motif can be predicted by sensitive sequence analysis methods (FFAS) to possess the structures of zincin-like metalloproteases (see Table 1). Thus, zincin-like fold can be predicted for eight structurally uncharacterised Pfam domains possessing the HExxH motif. One of them is the fatty acid desaturase domain (FA_desaturase, PF00487) that is present in proteins coded by eight human genes [44], [45]. The remaining seven are DUF462, DUF922, DUF1025, DUF2248, DUF2342, DUF3267 and SprA-related (PF12118) domains. Metalloprotease function is most likely conserved in these families, as judged from the conservation of active site motifs (see Fig. 2).

**Table 1:** Structure predictions for domain families with majority of members possessing the HExxH motif.

Query	% of domain family members possessing the HExxH motif	FFAS *Z-score*	% sequence identity	top FFAS hit	First prediction of a zincin-like structure
CLCA_N (PF08434) Calcium-activated chloride channel	72	−6,3	11	d1kufa_ d.92.1.9 (A:) Snake venom metalloprotease [Trimeresurus mucrosquamatus], atrolysin E	[28]
SprA-related (PF12118) SprA-related family	95	−7,7	11	PF05569.4; Q8RPJ4_DESHA/7–280; BlaR1 peptidase M56	this article
FA_desaturase (PF00487) Fatty acid desaturase	37	−8,4	16	d1k7ia2 d.92.1.6 (A:18–258) Metalloprotease [Erwinia chrysanthemi]	this article
Metallopep (PF12044) Putative peptidase family	87	−12,3	20	d1c7ka_ d.92.1.1 (A:) Zinc protease [Streptomyces caespitosus]	Pfam annotation
MtfA (PF06167) Phosphoenolpyruvate:glucose-phosphotransferase regulator	100	−43,9	11	d1j7na2 d.92.1.14 (A:551–773) Anthrax toxin lethal factor, N- and C-terminal domains [Bacillus anthracis]	[7]
DUF2248 (PF10005) Uncharacterized protein conserved in bacteria	100	−13,9	10	d1j7na2 d.92.1.14 (A:551–773) Anthrax toxin lethal factor, N- and C-terminal domains [Bacillus anthracis]	this article
DUF2265 (PF10023) Predicted aminopeptidase	100	−7,7	11	d3b7sa3 d.92.1.13 (A:209–460) Leukotriene A4 hydrolase catalytic domain [Homo sapiens]	Pfam annotation
DUF462 (PF04315) Protein of unknown function	100	−7	13	d1j7na2 d.92.1.14 (A:551–773) Anthrax toxin lethal factor, N- and C-terminal domains [Bacillus anthracis]	this article
DUF922 (PF06037) Bacterial protein of unknown function	100	−7,4	10	d1kjpa d.92.1.2 (A:) Thermolysin [Bacillus thermoproteolyticus]	this article
DUF3267 (PF11667) Protein of unknown function	83	−10,8	12	d1asta_ d.92.1.8 (A:) Astacin [Astacus astacus)]	this article
DUF1025 (PF06262) Domain of unknown function	76	−43,4	34	d3e11a1 d.92.1.17 (A:1–113) Uncharacterized protein Acel_2062 [Acidothermus cellulolyticus]	this article
DUF2342 (PF10103) Uncharacterized conserved protein	43	−93,8	19	d3cmna1 d.92.1.16 (A:43–391) Uncharacterized protein Caur0242 [Chloroflexus aurantiacus]	this article

### Substituted HExxH sites in metalloproteases

Both among the HExxH proteins that are characterised as metalloproteases and those predicted as such, many domains (approximately half of the cases) have active site motifs occasionally “broken down”, i.e. with substitutions at one of the critical positions, His, Glu or second His, see the protein families with less than 100% of conserved HExxH motif in Fig. 1. The domain families with significant fraction of substituted active sites occur in all domains of life, bacteria, eukaryotes and archaea alike.

Interestingly, the substituted motifs exhibit non-random substitution patterns (see Fig. 2). Both the first and the second histidine residues are significantly more often than expected by chance replaced with positively charged arginine and lysine. Generally in protein sequences, histidine is most often replaced by glutamine. The glutamate residue of the HExxH motif in the zincin-like proteins is most often replaced by glutamine, as generally in proteins. The biological role of lysine- or arginine-replaced histidines in the substituted active sites are not clear since these positively charged residues are not expected to participate in zinc ion binding. The replacement frequencies of the critical histidine and glutamate residues in substituted HExxH proteins deviate largely from the general replacement frequencies observed in proteins for these residues.

Several substitutions do occur in HExxH motifs at least twice as often as in proteins in general (see Table S1). The first histidine residue of the motif is approximately twofold more often than in an average protein substituted by Glu or Arg. The second histidine residue is also approximately twofold more often replaced with Lys or Arg and almost fivefold more often - by Leu. The catalytic Glu is very often replaced by Ala, Leu and Gln (2-, 3- and 3-fold, respectively). These substitutions can be divided into two categories: first, missense mutations resulting from a single-nucleotide change in a codon: H→R, H→Q, H→L and E→Q, and second, amino acid substitutions requiring two nucleotide changes in the corresponding codon: H→K, H→E, E→L.

The common active site substitutions are not spread evenly among the HExxH metallopeptidase families (see Fig. 2). The H1→R change (substitution of first histidine of the motif by an arginine) is found often in CLCA_N peptidases and in BSPs (Basic Secretory Proteins). The H1→E change is found often in peptidases M2. The H5→L change occurs in reprolysins, while the H5→K change – in CLCA_N peptidases and reprolysins. The substitution E2→Q occurs in peptidases M54 and in BSPs (Basic Secretory Proteins) and the change E2→L is seen in reprolysins. Interestingly, only some of the unusually frequent active site substitutions are biochemically conservative, retaining the hydrophilic/charged properties of a residue (e.g. H→R, H→K, E→Q). Intriguingly, other frequent substitutions do change strongly the properties of the amino acid residue from hydrophilic and/or charged to hydrophobic (H→L, E→L). Thus, probably active site substitutions observed in HExxH proteins are driven by more than one biological mechanism. Some substitutions may allow a metalloprotease to partly retain its biochemical properties (e.g. zinc ion binding) while other changes may definitely abrogate the original activity.. The roles of inactive HExxH metalloproteases are not well-understood. The best studied are some of the mammalian ADAM family members that have HExxH substitutions such as LQxxL or HQxxH, and are involved in sperm-egg interactions in the fertilisation process [46], [47]. Although the molecular function of the probably inactive proteases is mysterious, they can be expected to act as decoys, mimmicking other, proteolytically active ADAM paralogues, or as accessory proteins to their active counterparts.

### The CLCA family

The CLCA proteins, defined herein as those possessing the CLCA_N peptidase domain (PF08134) are one of the zincin-like metalloprotease families identified in this study as having the active site motif substituted in a number of cases (see Fig. 2).

**Figure 3:**
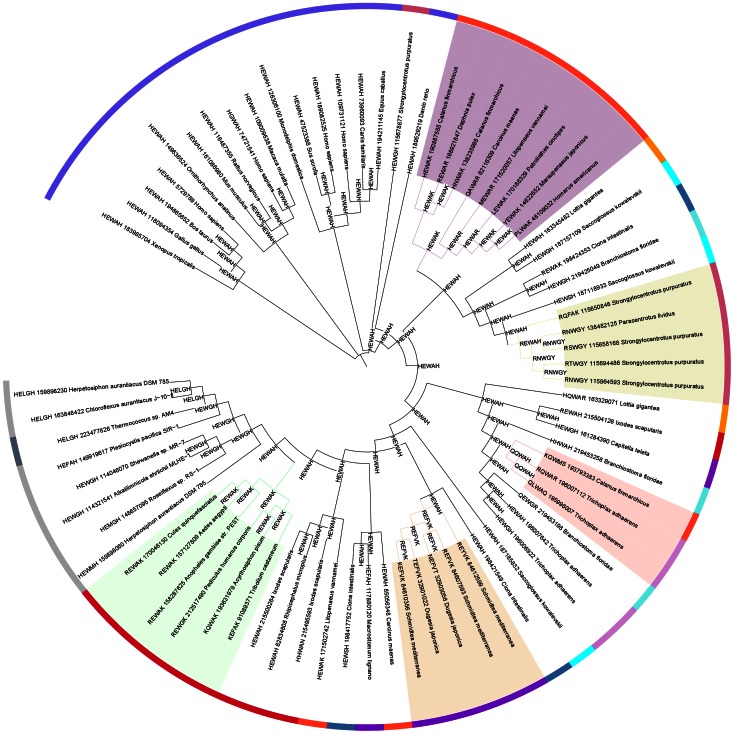
Phylogenetic tree (ANCESCON, see Methods) of selected representatives of the CLCA_N domain. Locations of proteins with substituted and correct active site motifs. Also predicted active sites of ancestral sequences shown.

A survey of CLCA_N domains shows widespread presence throughout *Metazoa* including early branching Plocozoan (*Trichopla*x), with notable absence in some model organisms like *Drosophila* or *Caenorhabditis* [28]. No CLCA_N domains were found in other eukaryotic taxa including *Fungi*, plants or amoebae. An analysis of distribution of substituted and correct active site motifs in a phylogenetic tree of selected representatives of the CLCA_N domain suggests that loss of proper active site in the CLCA family occurred most likely independently five times in specific lineages (see Fig. 3) rather than once in an ancestral CLCA_N domain. Despite multiple occurrences of CLCA proteins in various organisms, they often represent lineage-specific expansions, e.g. human and Plocozoan multiple CLCAs all originate from single proteins specific to their respective lineages (see Fig. S1).

**Figure 4:**
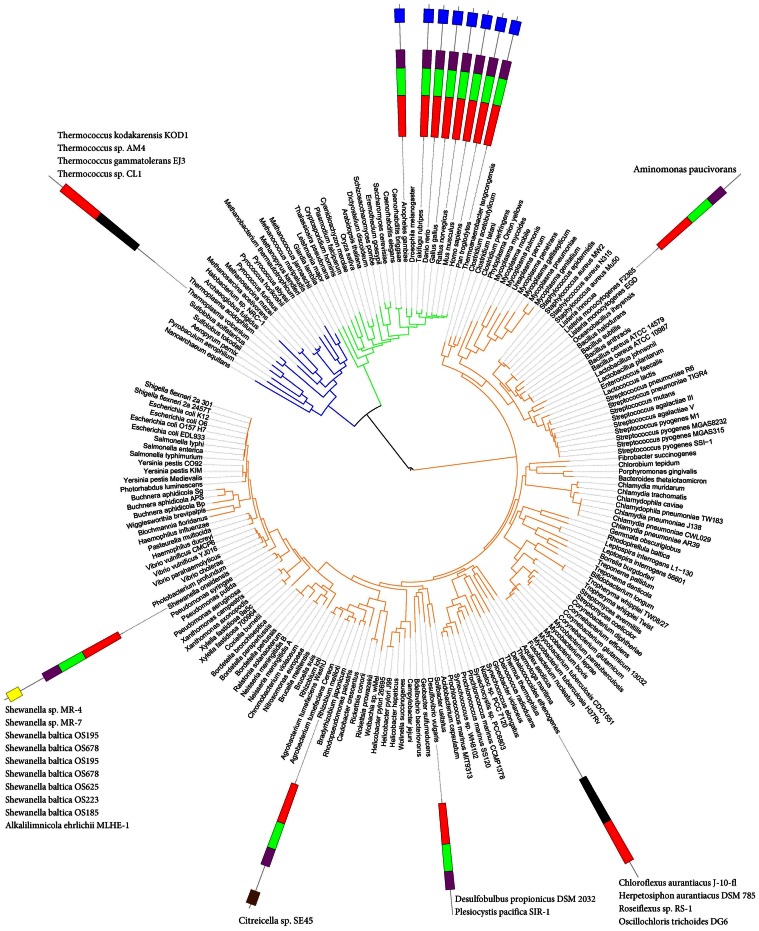
Tree of Life, i.e. representative species tree (adapted from iTOL [77]), with approximate locations of CLCA protein-possessing organisms shown. Schematic diagrams of domain architectures shown also: red, CLCA_N; green, von Willebrand factor type A; magenta, DUF1973; blue, fibronectin type III; black, other.

Interestingly, CLCA_N domains were also found in a number of prokaryotic genomes, including one archaeal genus (*Thermococcus*) and a handful of bacterial strains from several bacterial phyla. Strikingly, the CLCA_N-possessing strains are scattered throughout several main bacterial phyla (see Table 2 and Fig. 4). Thus, CLCA proteins are found in *Chloroflexi*, *Synergistetes*, alpha-, gamma- and delta-proteobacteria, but not in more than three five strains from each phylum.

**Table 2:** Habitats and lifestyles of bacteria and archaea possessing CLCA proteins.

Species, strain	phylum	environment	lifestyle	oxygen requirement	energy source	thermo-philic
*Alkalilimnicola ehrlichii MLHE-1*	γ-proteobacteria	aquatic	free living	facultative anaerobic	chemoautotroph	−
*Aminomonas paucivorans*	*Synergistetes*	aquatic/sewage	free living	anaerobic	chemoautotroph	−
*Chloroflexus aurantiacus J-10-fl*	*Chlorflexi*	aquatic/hot springs	free living	facultative aerobic	photoautotroph	+
*Citreicella sp. SE45*	α-proteobacteria	aquatic	free living	aerobic	chemoautotroph	−
*Desulfobulbus propionicus DSM 2032*	δ-proteobacteria	aquatic/marine sediments	free living	anaerobic	chemoautotroph	−
*Herpetosiphon aurantiacus DSM 785*	*Chlorflexi*	aquatic	free living	aerobic	chemotroph	−
*Plesiocystis pacifica SIR-1*	δ-proteobacteria	marine/aquatic	free living	aerobic	chemoheterotroph	−
*Roseiflexus sp. RS-1*	*Chlorflexi*	aquatic	free living	aerobic	phototroph	+
*Shewanella sp. MR-4*	γ-proteobacteria	aquatic	free living	facultative anaerobie	heterotroph	mesophile (30–40°C)
*Thermococcus_sp._AM4*	*Euryarchaeota (Archaea)*	aquatic	free living	facultative anaerobie	chemoautotroph	hyper-thermofile

**Figure 5:**
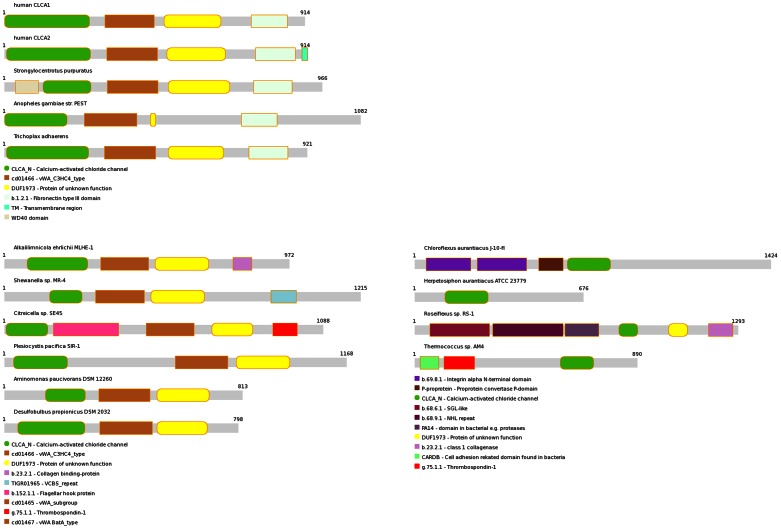
Protein domain architectures (Pfam) of selected CLCA proteins.

The typical domain architecture of a metazoan CLCA protein, as shown in Figs. 4 and 5, is repeated in bacterial homologues from alpha-, gamma- and delta- Proteobacteria, as well as *Synergistetes*. Other prokaryotic CLCA proteins (archaeal and those from *Chloroflexi*) have the CLCA_N domain thrown into a different, species-specific domain architecture (see Fig. 5). Interestingly, the typical CLCA domain architecture is also present in two species, the gamma-proteobacterium *Teredinibacter* and the euryarcheon *Methanosarcina*, with the CLCA_N domain replaced by two unrelated enzymatic (peptidase) domains, Peptidase_M10 and CHAP, respectively [22], [48].

**Figure 6:**
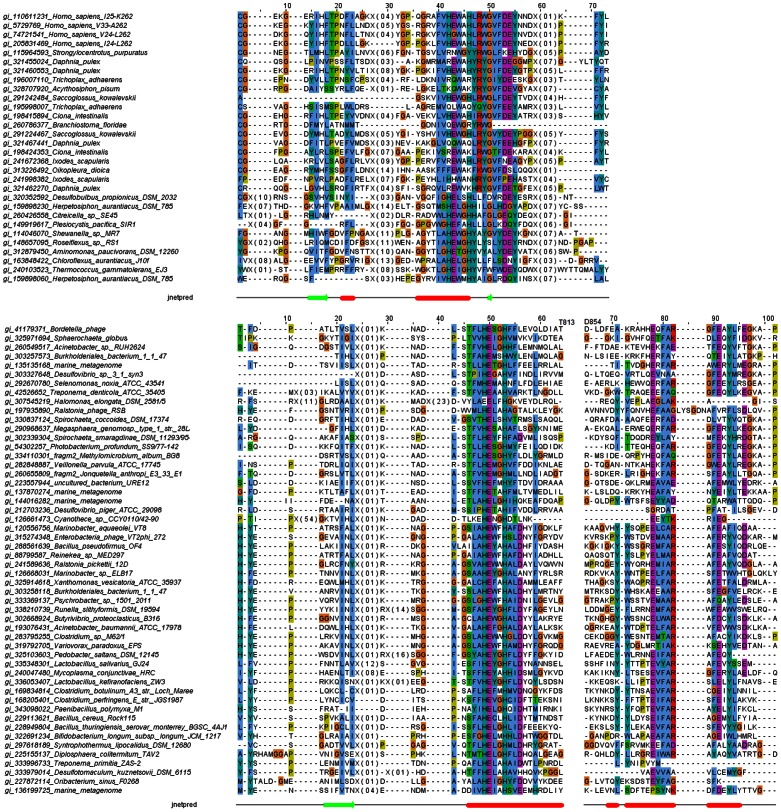
Multiple sequence alignments of representative CLCA_N sequences (top) and representative CLCA_X sequences (bottom). Only regions around the predicted HexxH active site shown. Predicted secondary structures shown (jnetpred). Full versions of the alignments shown in Figs. S2 and S4, respectively.

Conservation of the active site motif and general sequence conservation in the CLCA_N family suggests its peptidase function is broadly conserved (see the multiple alignment in Fig. 6, top, and Fig. S2). The cysteine-rich domain following the CLCA_N core metalloprotease domain (approx. residues 200–260 in human CLCA1 protein, see the multiple alignments) has no detectable homologues in known proteins and may be hypothesized to be involved in stabilisation of the peptidase domain and/or substrate binding [31].

The CLCA_N domains of the bacterial CLCA proteins are grouped together in a typical phylogenetic tree of the CLCA_N domains from different organisms (see Fig. 3), however, their precise phylogenetic relation to metazoan CLCA_N domains, hence their origin, remains unclear. The variability of sequences in the family precludes a reliable phylogenetic tree that could suggest the origin of the putative HGT event.

It is argued that phylogenetic analysis using DNA sequences, using the codon alignment derived from protein sequence alignment can be more reliable than the corresponding analysis using only protein sequences [49]. In the Fig. S3, two CLCA metalloprotease domain phylogenetic trees are presented side-by-side: one derived from mRNA sequences and the other derived from protein sequences, both built using the same protein sequence alignment. The nucleotide sequence tree has generally much better bootstrap values. The trees are generally similar in grouping sequences from major Metazoan taxa (e.g. vertebrates, insects, crustaceans), however they differ in placement of several highly diverged sequences that may have been subject to specific evolutionary pressures. Also, the two trees differ in placement of prokaryotic sequences whereas only the protein sequence tree groups all the prokaryotic sequences together. However, the low bootstrap values even for the nucleotide sequence tree preclude a reliable elucidation of the origin of the putative HGT of CLCA domain from Metazoa to prokaryotes. Also, because of low bootstrap values and the presence of highly divergent sequences (e.g. the lower branches in Fig. S3, upper part), the molecular clock is not applicable here for estimation of time of the likely horizontal gene transfer event recorded in the tree,

The habitats of CLCA_N domain-possessing prokaryotic strains are strikingly similar. These organisms are all aquatic and free living, usually aerobic, however they differ in temperature preferences and energy sources used (see Table 2). Taking together the taxonomic spread of CLCA proteins in prokaryotes and the conservation of their multi-domain composition, the most plausible evolutionary scenario seems to be that of horizontal gene transfer from eukaryotes (*Metazoa*) to bacteria. This direction of the transfer is most likely because of the ubiquity of CLCAs in *Metazoa* and their paucity in prokaryotes. Of note, an automated approach for detection of phylogenetically atypical genes in has identified a CLCA homologue from *Shewanella* as a HGT candidate [50]. Because all the CLCA-possessing organisms live in aquatic environments, it may be hypothesised that the horizontal gene transfer of a metazoan CLCA gene to bacterium occurred in an aquatic milieu.

Another argument in favour of a HGT scenario could have been conservation of CLCA genomic neighbourhoods in prokaryotes, however, no such conservation can be observed here. Genomic neighbourhood similarities are usually restricted to the species level. However, protein families annotated with some common functional themes can be found in the neighbourhoods, e.g. protease genes (COG1988, predicted membrane-bound metal-dependent hydrolases and COG4955, distant caspase homologues). Also, present in the neighbourhoods are COG2199 (diguanylate cyclase with PAS/PAC sensor), COG VicK (histidine kinase), COG Baes (sensor histidine kinase), COG3899 (sensor histidine kinase), COG-NtrB (signal transduction histidine kinase, nitrogen specific). The protease and signalling domains present in bacterial CLCA neighbourhoods are reminiscent of the vertebrate CLCA extracellular protease functions, including the regulatory functions.

A homologous gene acquired by a host by the way of HGT and whose evolution therefore does not match the evolution of its host organism has been termed xenologue [51]. The hypothetical eukaryote-to-bacteria gene transfer described here is obviously not the first known case of eukaryote-derived xenologues in bacterial genomes. Recently, three-dimensional structures of two virulence factors from *Bacteroidetes* bacteria have been solved. Both three-dimensional structures turned out to be metalloproteases. Their structural features and sequence similarity relationships strongly suggested these proteins had been acquired from mammals by a bacterial pathogen [52], [53]. Although the prevalence of HGT between eukaryotes and prokaryotes has only recently been appreciated [54], its crucial importance for the evolution of bacteria, archaea and viruses has been known for a decade and HGT is now established as one of key mechanisms modulating the classic Darwinian mechanisms of evolution [51], [55], [56].

### The CLCA_X (CLCA-like) family

**Figure 7:**
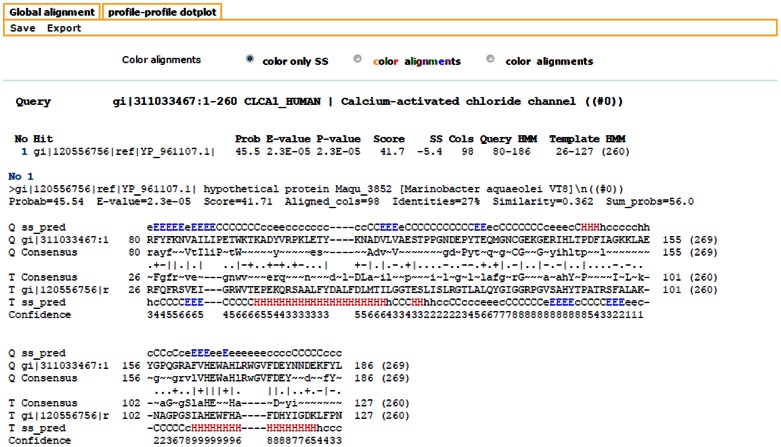
HHalign alignment between HHsenser-generated profiles of CLCA_N and CLCA_X protease domains.

Among the distant subsignificant hits in PSI-BLAST sequence similarity searches for CLCA proteins, a recurring sequence could be noted: a 260 residue-long protein annotated as hypothetical protein Maqu_3852, NCBI gi:120556756, from gamma-proteobacterium *Marinobacter aquaeolei* VT8. It turned out to be a founding member of a large group of bacterial and viral proteins, termed herein CLCA_X. In the Pfam database, most of these could be assigned to the uncharacterised Pfam-B_1042 domain family. Similarity between CLCA_N protease domain and CLCA_X is significant, as proved by HHalign alignment between HHsenser-generated profiles of the two groups (alignment p-value 2.3E-05, see Fig. 7).

Charting the CLCA_X family using HHsenser brought 153 proteins, after CD-hit clustering at 95 sequence identity. Clustering at 70% sequence identity yielded 115 representative sequences. A substantial part of these, were annotated as viral proteins, in general, only 12 out of 115 proteins did not hit any mobile genomic elements (phage, prophage or plasmid sequences) in the ACLAME database (see list in Table S2).

For groups of distantly related protein families, phylogenetic trees are in general not feasible. The approximate topology of sequence similarity networks can be visualized by graph approaches utilising sets of pairwise similarities, e.g. CLANS [57]. In order to locate the CLCA_N and CLCA_X families within the context of zinc dependent metalloproteases of the zincin fold, we applied the CLANS clustering algorithm to the complete set of families of the Peptidase_MA clan and several more families that were identified as related to them (see Results, first section).

**Figure 8:**
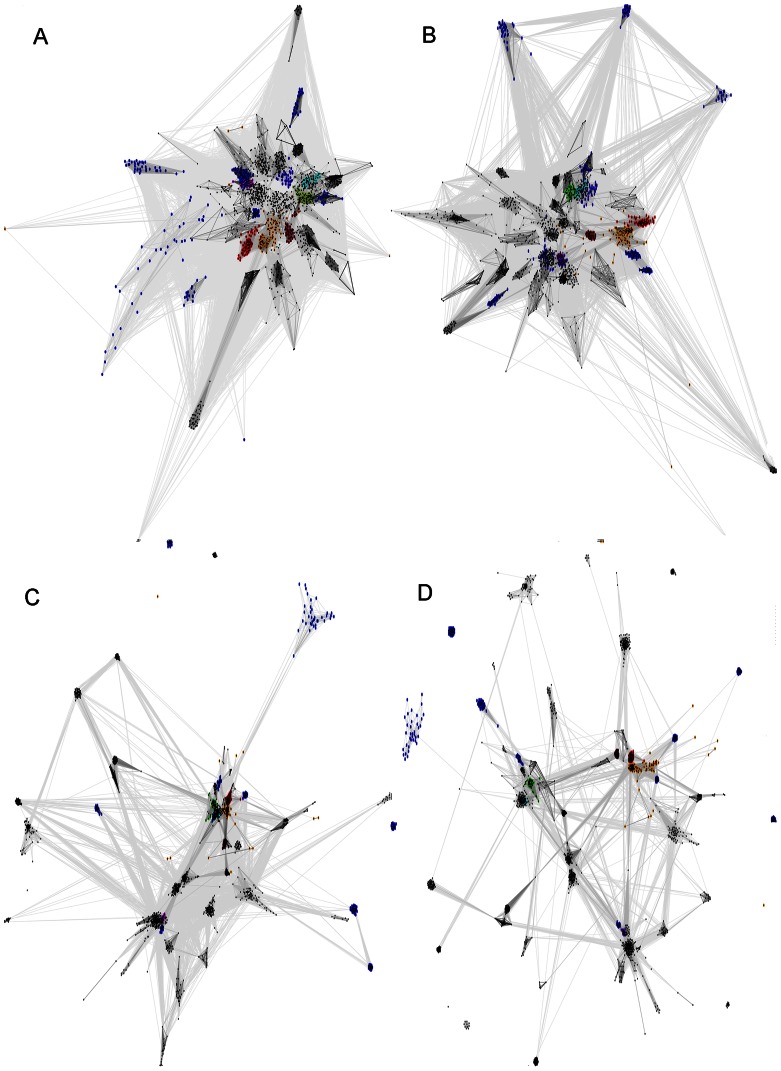
CLANS sequence similarity network for zincin-like proteins. Pfam clan Peptidase_MA and other zincin-like proteins included (see Methods). Four different BLAST E-value thresholds used for CLANS clustering. Pfam clan Peptidase_MA proteins: Matrixin (Peptidase_M10): green, Peptidase_M1: magenta, Reprolysin: cyan, Zn_peptidase_2: brown. Others in the Peptidase_MA clan: black Proteins not included in the Pfam clan Peptidase_MA: CLCA_N: red, CLCA_X: orange, Others outside the Peptidase_MA clan: blue. A) Relations with significance of P-value below 0.1, B) P-value below 0.01, C) P-value below 1E-5 and D) P-value below 1E-10.

In the CLANS clustering graph, the CLCA_X group locates consistently as a sister group to CLCA_N (see Fig. 8). Even using various significance thresholds for the CLANS analysis, one obtains a consistent picture whereas CLCA_X is clustered together with CLCA_N and close to the central zincin-like families. The known protease family closest to CLCAs is the Peptidase_M64 (IgA peptidase) [58], a secreted protease present in many bacterial strains that have humans as hosts, including pathogenic bacteria. Then, the next Pfam family most similar to CLCA_X was PF04298 Zn_peptidase_2, an uncharacterized bacterial family, present mostly in *Firmicutes* and *Bacteroidetes*. Further, relatively closely to CLCA_X occurred the mostly bacterial uncharacterised family DUF955, and the ubiquitous Peptidase_M3 family of secreted proteases (e.g. neurolysins) present in prokaryotes and eukaryotes alike, including humans [59], [60]. Among the above-mentioned metalloprotease families, those characterized (Peptidase_M64, Peptidase_M3 and CLCA_N) are known to act as secreted proteases.

CLCA_X proteins are found in several bacterial phyla, partly those that possess CLCA proteins with CLCA_N domains, namely alpha-, beta-, gamma-, delta-proteobacteria, as well as *Synergistetes*, *Spirochaetes* and *Firmicutes.* Also, *Caudovirales* viruses possess CLCA_X proteins. However, CLCA_N and CLCA_X domains are not found in the same species with the exception of *Shewanella* sp. MR-7. The sequence conservation within the CLCA_X family and similarity to CLCA_N domains suggests a conserved protease function and similar active site architectures.

The CLCA_X active site conforms to a consensus HExxHxxxD, only somewhat similar to the CLCA consensus, which is HExxHxxxGxxDEY (see Fig. 6, bottom, and Fig. S4), whereas either the aspartate or the second glutamate residue in the latter motif has been proposed as a likely additional ligand of the zinc ion [31]. The conserved aspartate in the CLCA_X active site motif may perform the same role. The CLCA active site motif is remarkably similar to the active site of known Peptidase_M64 proteins, the HExxHxxxxLxDEY motif [58], [61]. In a recently solved structure of a Peptidase_M64 metalloprotease (PDB code 3P1V), a second zinc ion is present, liganded by E of the conserved DEY motif, and by three strongly conserved cysteines, located 90–100 residues away from the HExxH motif towards the C-terminus. Thus, such a role for the DEY motif in the CLCA_N can be postulated.

Although many CLCA_X proteins are long (e.g. more than half of the CLCA_X proteins are longer than 500 residues, more than sixty are 1000 residues or longer), almost none of them contains any known protein domains, suggesting possible existence of completely novel protease auxiliary domains.

## Conclusions

The CLCA proteins receive continued attention due to their medical and biological relevance [62]–[64]. The details of the catalytic metalloprotease activity of CLCA are being elucidated, as well as mechanisms of ion channel activation [31].

Here, we argue for an atypical evolutionary scenario of HGT, from multicellular eukaryotes to bacteria and archaea. Such transfers have been described previously, yet they involved *Wolbachia*, intracellular parasites of *Drosophila* [54], [65]. A CLCA protein from the bacterium *Shewanella* has been identified previously as a putative HGT gene [50]. Although the horizontal gene transfer of CLCA genes is only a hypothesis, it seems to be the best explanation of the phylogenetic CLCA distribution observed. Thus, study of distant prokaryotic homologues of CLCA may shed light on its biological functions in *Metazoa*, including humans.

We also argue that the current catalogues of proteins families, including enzymes, are still incomplete and insufficient, as shown by our metalloprotease structure and function prediction for eight uncharacterised Pfam families (see Table 1). The number of HExxH motifs and HExxH motif-containing families suggests that other HExxH metalloprotease families may remain undicovered. We also expect that prevalence and functional importance of inactive homologues of known enzymes may be under-appreciated.

## Methods

### Survey of HExxH proteins

The Trembl database, version 04.2010, counting 10706472 sequences was used, and the HExxH proteins were extracted using PrositeScan [66]. The proteins with motifs identified were screened against the Pfam database (version 24.0) [67]. Fold predictions for HExxH rich domain families were performed using the FFAS server [68], [69].

The CLANS algorithm [57] was run with five iterations of PSI-BLAST, using the BLOSUM45 substitution matrix and inclusion threshold of 0.001 on nr90 and env90 sequence databases. For the graphs, PSI-BLAST similarity relations with significance of P-value below 0.1 were considered, alternatively, thresholds of 0.01, 1E-5 and 1E-10 were used. The following protein families were included in the analysis: 52 families belonging to the Pfam Peptidase_MA clan, and also 12 families identified herein as similar to zincins (see Table 1): CLCA_N (PF08434), SprA-related (PF12118), FA_desaturase (PF00487), Metallopep (PF12044), MtfA (PF06167), DUF2248 (PF10005). DUF2265 (PF10023), DUF462 (PF04315), DUF922 (PF06037), DUF3267 (PF11667), DUF1025 (PF06262), DUF2342 (PF10103), and finally the CLCA_X family described herein.

### Survey of the CLCA_N homologues. Building representative sets of CLCA_N and CLCA_X sequences

The following sequences were used as seeds for five separate HHsenser [70] searches: residues 1–260 of human CLCA1 (gi|311033467), residues 1–312 of putative outer membrane adhesin like protein from *Shewanella* sp. MR-4 (gi|113971723), residues 575–875 of unnamed protein product from *Spirochaeta coccoides* DSM 17374 (gi|330837124), full length sequence (260 residues) of hypothetical protein Maqu_3852 from *Marinobacter aquaeolei* VT8 (gi|120556756), residues 700–1000 of Bbp10 protein from *Bordetella* phage BPP-1 (gi|41179371). Results from the first two and last three searches were combined into CLCA_N and CLCA_X sequence sets. The sets were cleared of redundant entries using the CD-HIT program [71] at 95 and 70% sequence identity levels, creating thus full and representative sets. HHsenser was ran on combined nr and env_nr (environmental sequences) databases using standard parameters. The CLCA_N full and representative sequence sets contained 160 and 92 sequences, respectively, while the CLCA_X sequence sets contained 153 and 115 sequences, respectively.

The five HHsenser run seeds were significantly similar, as judged by the FFAS profile-profile algorithm [72]: CLCA1 human vs *Shewanella*: Zscore −56.3, 12% sequence identity over 255 residues; *Marinobacter* Maqu_3852 vs *Bordetella* phage BPP-1: Zscore −33.6, 13% sequence identity over 182 residues; *Marinobacter* Maqu_3852 vs *Spirochaeta gi|330837124*: Zscore −32.6, 13% sequence identity over 181 residues. Similarities of the CLCA_N and CLCA_X families were also confirmed by HHalign [73] using HHsenser-generated multiple sequence alignments for human CLCA1 and *Marinobacter* protein Maqu_3852 as input. HHalign alignment was significant (E-value 3E-05) and covered 98 residues (see Fig. 7).

### Phylogenetic tree reconstruction

A phylogenetic tree of the metazoan CLCA_N domains was built in order to establish the origin of CLCA_N domains with substituted active sites. For preparation of multiple sequence alignment, sequences of CLCA_N domains from various groups of organisms vertebrates, invertebrates and *Prokaryota* were manually selected. These sequences possessed both the correct and substituted active sites. The T-coffee [74] program with standard parameters was used to build the alignment. The obtained alignment was refinement using the G-blocks algorithm [75] to eliminate poorly aligned positions and divergent regions. The G-blocks option allow less strict flanking positions was used. The refined alignment was used to create phylogenetic tree using the ANCESCON program [76]. This algorithm provided reconstructed sequences for the tree root and all the internal nodes. The maximum likelihood method for estimaion of substitution rate factors was applied for estimation of the likelihood of residues at a site given a tree.

### Other methods

For visualization of the reconstructed phylogenetic tree, the *on-line* tool iTOL [77] was applied. The sequence logos were created using the aligned Pfam seed sequences for the protein domains studied and the Weblogo tool, weblogo.berkeley.edu [78].

Identification of similarities to phage and viral proteins was performed using Blast queries on the ACLAME database [79].

The aminoacid substitution frequencies of residues in the HExxH motif were compared against the corresponding frequencies observed generally in proteins, as encoded in the PAM250 matrix [80]. Protein domains were identified using the Pfam database Pfam HMM tool [42].

## Supporting Information

Figure S1 Phylogenetic tree of human and Plocozoan CLCA_N domains.
(PDF)

Figure S2 Multiple sequence alignment of the full sequence set of CLCA_N domains (see Methods). Full version of upper part of Fig. 6.
(PDF)

Figure S3 Phylogenetic tree (PhyML, see Methods) of selected representatives of the CLCA_N domain. Upper part: tree built using nucleotide sequences. Lower part: tree built using protein sequences. Both trees were built starting from the same protein sequence alignment. Branches with bootstrap values above 50% shown in green, Human sequences highlighted in blue, prokaryotic ones highlighted in red.
(PDF)

Figure S4 Multiple sequence alignment of the full sequence set of CLCA_X domains (see Methods). Full version of lower part of Fig. 6.
(PDF)

Table S1 Replacement frequencies of the critical H and E site residues in substituted HExxH motifs in the domains of the Peptidase_MA clan as defined in the Pfam database divided by corresponding replacement frequencies in proteins in general (as derived from the PAM250 substitution matrix). First column: replacement position within the HExxH motif. Values above 2 or below 0.5 in bold.
(DOC)

Table S2 Plasmid, virus and prophage BLAST hits for CLCA_X proteins (QueryID) obtained in the ACCLAME database.
(XLS)
