# Supplementary figures and images for: CLCAs - A Family of Metalloproteases of Intriguing Phylogenetic Distribution and with Cases of Substituted Catalytic Sites
Source: PLoS One. 2013 May 9;8(5):e62272. doi: 10.1371/journal.pone.0062272 (PMC3650047; doi:10.1371/journal.pone.0062272)

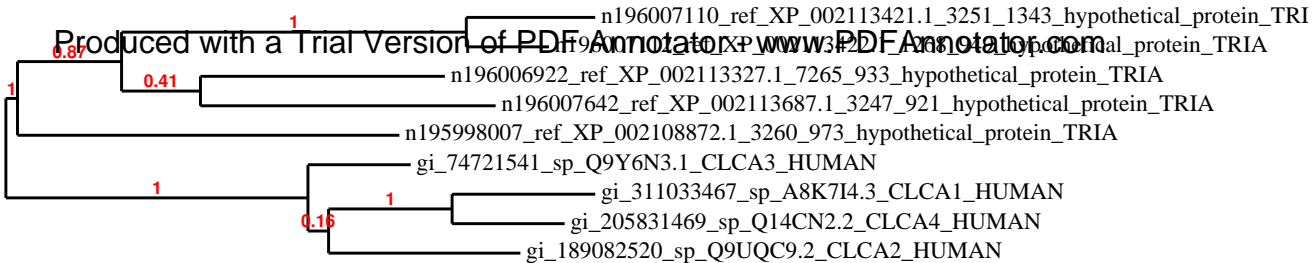

Suppl. Figure S1

Supplement: Figure S1 — Phylogenetic tree of human and Plocozoan CLCA_N domains. (PDF) [file pone.0062272.s001.pdf]

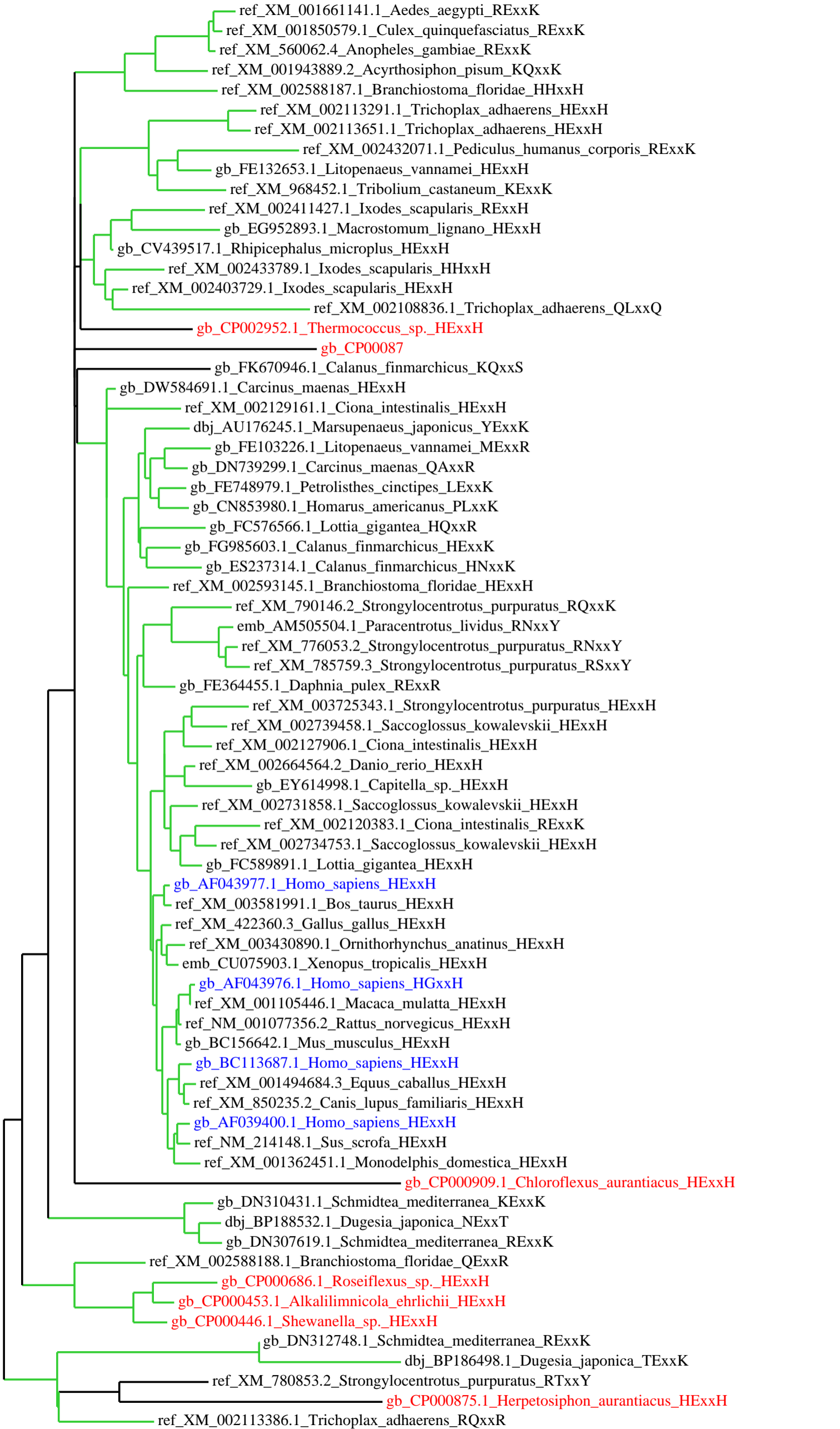

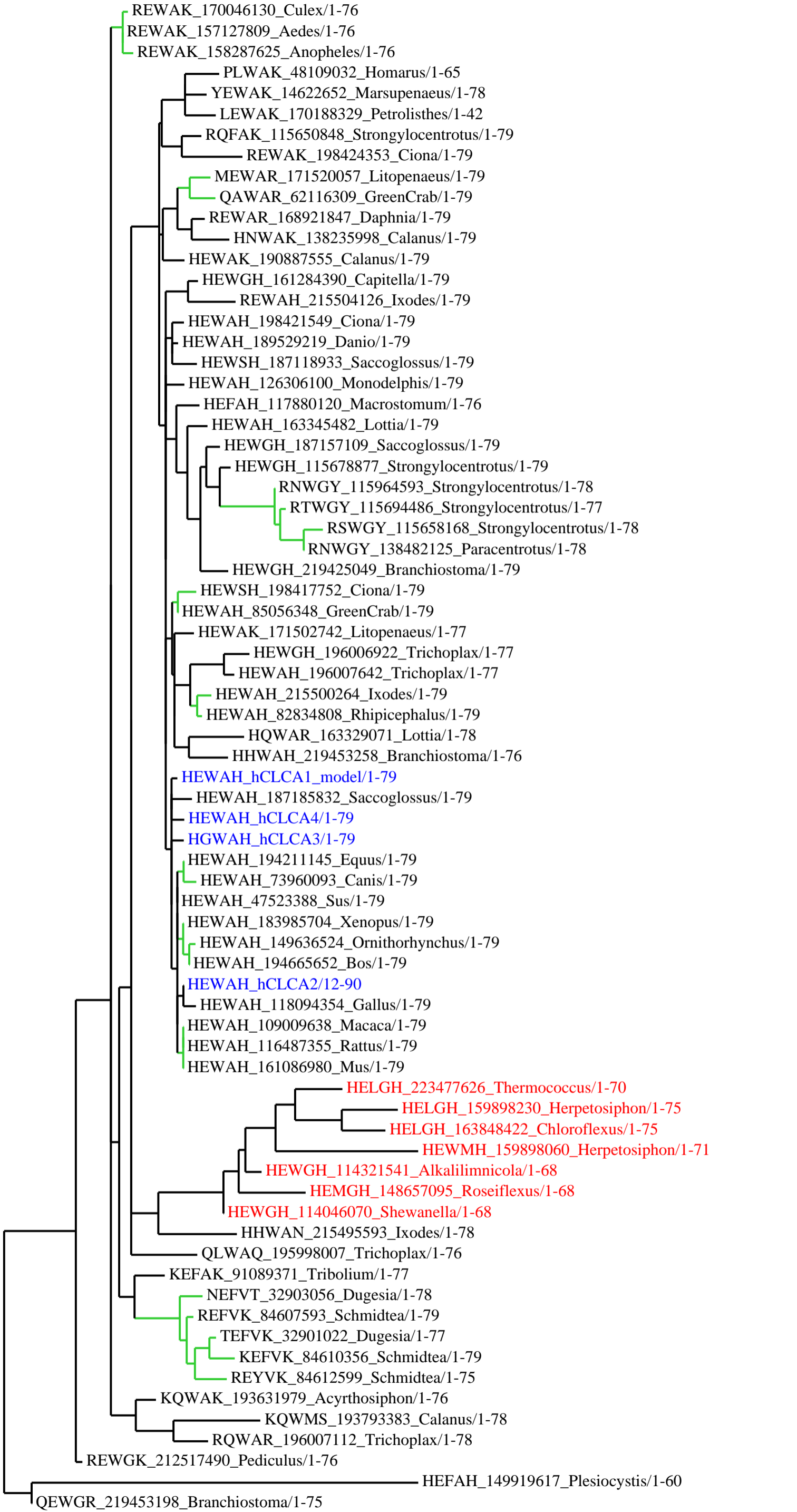

Supplement: Figure S3 — Phylogenetic tree (PhyML, see Methods) of selected representatives of the CLCA_N domain. Upper part: tree built using nucleotide sequences. Lower part: tree built using protein sequences. Both trees were built starting from the same protein sequence alignment. Branches with bootstrap values above 50% shown in green, Human sequences highlighted in blue, prokaryotic ones highlighted in red. (PDF) [file pone.0062272.s003.pdf]
