# Supplementary material for: CLCAs - A Family of Metalloproteases of Intriguing Phylogenetic Distribution and with Cases of Substituted Catalytic Sites
Source: PLoS One. 2013 May 9;8(5):e62272. doi: 10.1371/journal.pone.0062272 (PMC3650047; doi:10.1371/journal.pone.0062272)
Supplement: Figure S2 — Multiple sequence alignment of the full sequence set of CLCA_N domains (see Methods). Full version of upper part of Fig. 6. (PDF) [file pone.0062272.s002.pdf]

Produced with a Trial Version of PDF Annotator - www.PDFAnnotator.com

Suppl. Figure S2

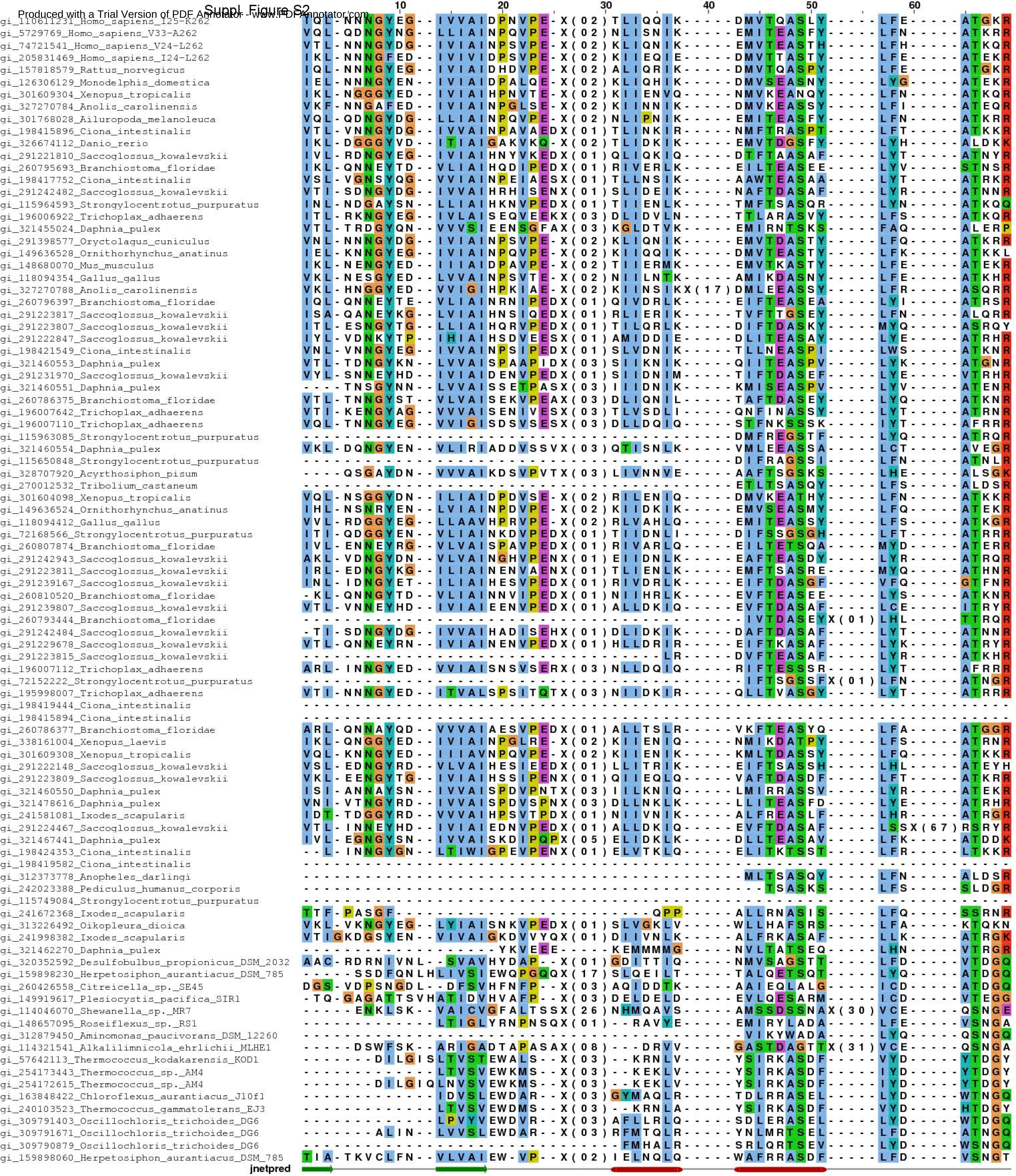

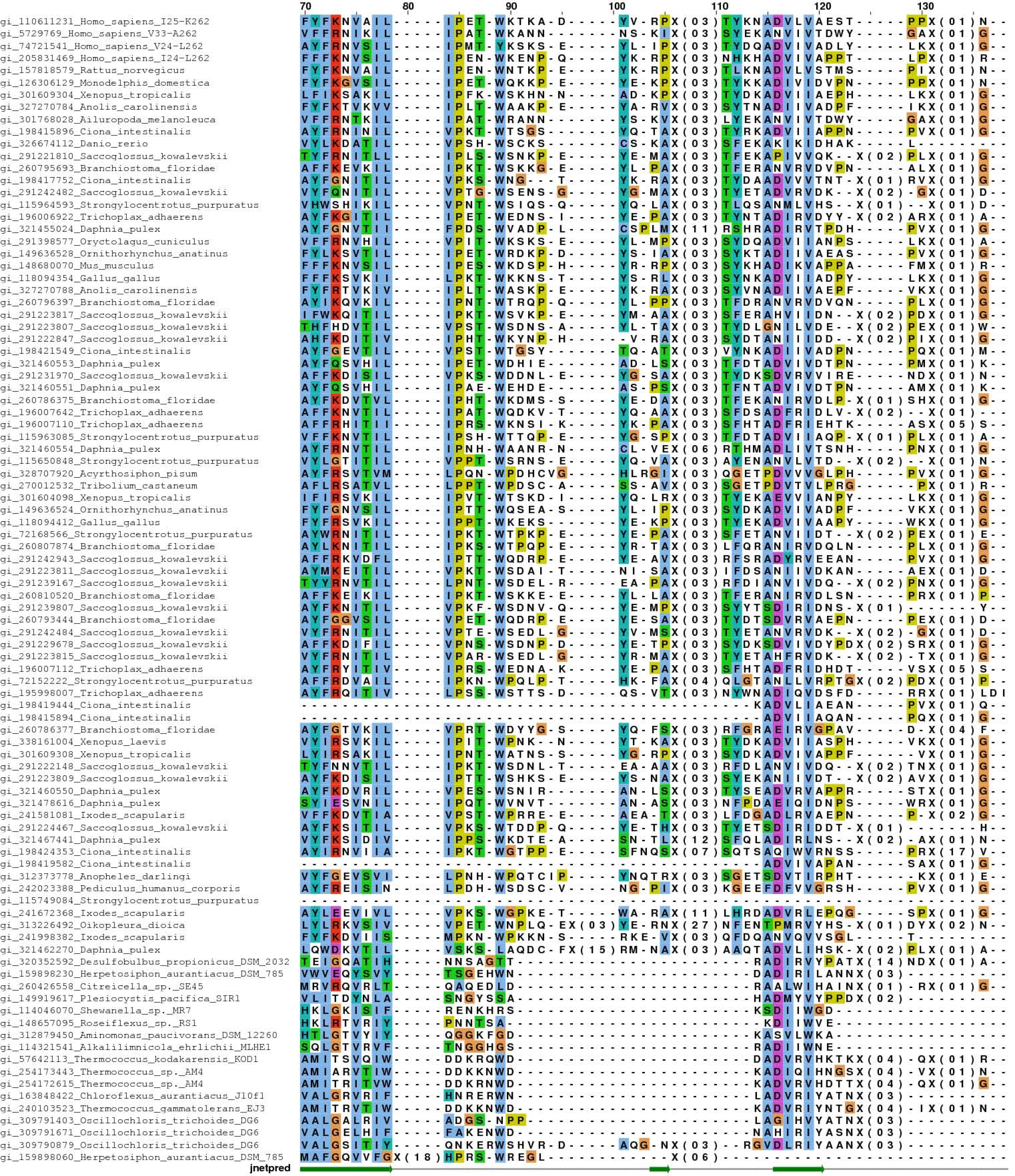
70
80
90
100
110
120
130
gi_110611231_Homo_sapiens_I25-K262
gi_5729769_Homo_sapiens_V33-A262
gi_74721541_Homo_sapiens_V24-L262
gi_205831469_Homo_sapiens_I24-L262
gi_157818579_Rattus_norvegicus
gi_126306129_Monodelphis_domestica
gi_301609304_Xenopus_tropicalis
gi_327270784_Anolis_carolinensis
gi_301768028_Ailuropoda_melanoleuca
gi_198415896_Ciona_intestinalis
gi_326674112_Danio_rerio
gi_291221810_Saccoglossus_kowalevskii
gi_260795693_Branchiostoma_floridae
gi_198417752_Ciona_intestinalis
gi_291242482_Saccoglossus_kowalevskii
gi_115964593_Strongylocentrotus_purpuratus
gi_196006922_Trichoplax_adhaerens
gi_321455024_Daphnia_pulex
gi_291398577_Oryctolagus_cuniculus
gi_149636528_Ornithorhynchus_anatinus
gi_148680070_Mus_musculus
gi_118094354_Gallus_gallus
gi_327270788_Anolis_carolinensis
gi_260796397_Branchiostoma_floridae
gi_291223817_Saccoglossus_kowalevskii
gi_291223807_Saccoglossus_kowalevskii
gi_291222847_Saccoglossus_kowalevskii
gi_198421549_Ciona_intestinalis
gi_321460553_Daphnia_pulex
gi_291231970_Saccoglossus_kowalevskii
gi_321460551_Daphnia_pulex
gi_260786375_Branchiostoma_floridae
gi_196007642_Trichoplax_adhaerens
gi_196007110_Trichoplax_adhaerens
gi_115963085_Strongylocentrotus_purpuratus
gi_321460554_Daphnia_pulex
gi_115650848_Strongylocentrotus_purpuratus
gi_328707920_Acyrthosiphon_pisum
gi_270012532_Tribolium_castaneum
gi_301604098_Xenopus_tropicalis
gi_149636524_Ornithorhynchus_anatinus
gi_118094412_Gallus_gallus
gi_72168566_Strongylocentrotus_purpuratus
gi_260807874_Branchiostoma_floridae
gi_291242943_Saccoglossus_kowalevskii
gi_291223811_Saccoglossus_kowalevskii
gi_291239167_Saccoglossus_kowalevskii
gi_260810520_Branchiostoma_floridae
gi_291239807_Saccoglossus_kowalevskii
gi_260793444_Branchiostoma_floridae
gi_291242484_Saccoglossus_kowalevskii
gi_291229678_Saccoglossus_kowalevskii
gi_291223815_Saccoglossus_kowalevskii
gi_196007112_Trichoplax_adhaerens
gi_72152222_Strongylocentrotus_purpuratus
gi_195998007_Trichoplax_adhaerens
gi_198419444_Ciona_intestinalis
gi_198415894_Ciona_intestinalis
gi_260786377_Branchiostoma_floridae
gi_338161004_Xenopus_laevis
gi_301609308_Xenopus_tropicalis
gi_291222148_Saccoglossus_kowalevskii
gi_291223809_Saccoglossus_kowalevskii
gi_321460550_Daphnia_pulex
gi_321478616_Daphnia_pulex
gi_241581081_Ixodes_scapularis
gi_291224467_Saccoglossus_kowalevskii
gi_321467441_Daphnia_pulex
gi_198424353_Ciona_intestinalis
gi_198419582_Ciona_intestinalis
gi_312373778_Anopheles_darlingi
gi_242023388_Pediculus_humanus_corporis
gi_115749084_Strongylocentrotus_purpuratus
gi_241672368_Ixodes_scapularis
gi_313226492_Oikopleura_dioica
gi_241998382_Ixodes_scapularis
gi_321462270_Daphnia_pulex
gi_320352592_Desulfobulbus_propionicus_DSM_2032
gi_159898230_Herpetosiphon_aurantiacus_DSM_785
gi_260426558_Citreicella_sp._SE45
gi_149919617_Plesiocystis_pacifica_SIR1
gi_114046070_Shewanella_sp._MR7
gi_148657095_Roseiflexus_sp._RS1
gi_312879450_Aminomonas_paucivorans_DSM_12260
gi_114321541_Alkalilimnicola_ehrlichii_MLHE1
gi_57642113_Thermococcus_kodakarensis_KOD1
gi_254173443_Thermococcus_sp._AM4
gi_254172615_Thermococcus_sp._AM4
gi_163848422_Chloroflexus_aurantiacus_J10fl
gi_240103523_Thermococcus_gammatolerans_EJ3
gi_309791403_Oscillochloris_trichoides_DG6
gi_309791671_Oscillochloris_trichoides_DG6
gi_309790879_Oscillochloris_trichoides_DG6
gi_159898060_Herpetosiphon_aurantiacus_DSM_785
jnetpred

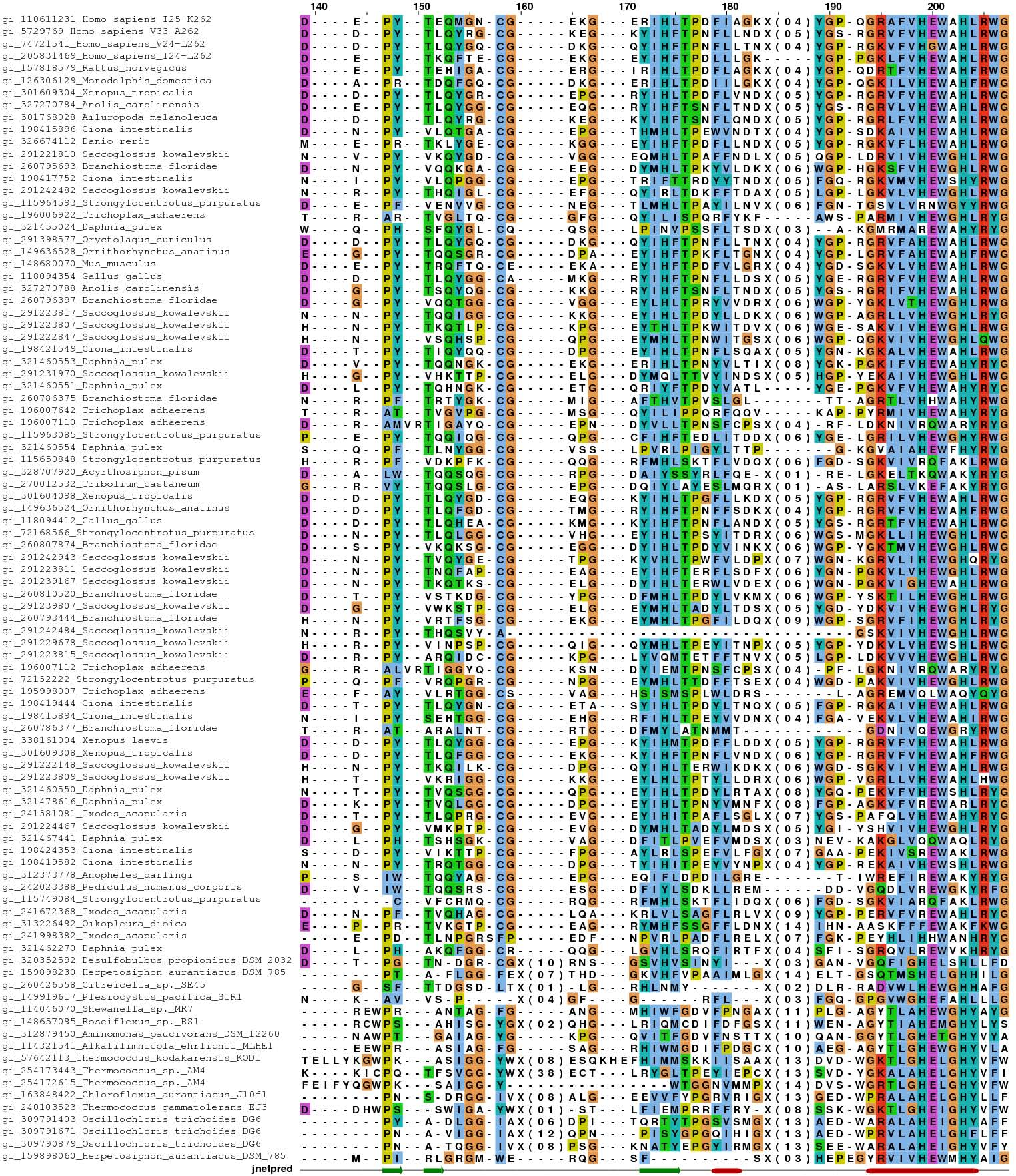

140
150
160
170
180
190
200
gi_110611231_Homo_sapiens_I25-K262
gi_5729769_Homo_sapiens_V33-A262
gi_74721541_Homo_sapiens_V24-L262
gi_205831469_Homo_sapiens_I24-L262
gi_157818579_Rattus_norvegicus
gi_126306129_Monodelphis_domestica
gi_301609304_Xenopus_tropicalis
gi_327270784_Anolis_carolinensis
gi_301768028_Ailuropoda_melanoleuca
gi_198415896_Ciona_intestinalis
gi_326674112_Danio_rerio
gi_291221810_Saccoglossus_kowalevskii
gi_260795693_Branchiostoma_floridae
gi_198417752_Ciona_intestinalis
gi_291242482_Saccoglossus_kowalevskii
gi_115964593_Strongylocentrotus_purpuratus
gi_196006922_Trichoplax_adhaerens
gi_321455024_Daphnia_pulex
gi_291398577_Oryctolagus_cuniculus
gi_149636528_Ornithorhynchus_anatinus
gi_148680070_Mus_musculus
gi_118094354_Gallus_gallus
gi_327270788_Anolis_carolinensis
gi_260796397_Branchiostoma_floridae
gi_291223817_Saccoglossus_kowalevskii
gi_291223807_Saccoglossus_kowalevskii
gi_291222847_Saccoglossus_kowalevskii
gi_198421549_Ciona_intestinalis
gi_321460553_Daphnia_pulex
gi_291231970_Saccoglossus_kowalevskii
gi_321460551_Daphnia_pulex
gi_260786375_Branchiostoma_floridae
gi_196007642_Trichoplax_adhaerens
gi_196007110_Trichoplax_adhaerens
gi_115963085_Strongylocentrotus_purpuratus
gi_321460554_Daphnia_pulex
gi_115650848_Strongylocentrotus_purpuratus
gi_328707920_Acyrthosiphon_pisum
gi_270012532_Tribolium_castaneum
gi_301604098_Xenopus_tropicalis
gi_149636524_Ornithorhynchus_anatinus
gi_118094412_Gallus_gallus
gi_72168566_Strongylocentrotus_purpuratus
gi_260807874_Branchiostoma_floridae
gi_291242943_Saccoglossus_kowalevskii
gi_291223811_Saccoglossus_kowalevskii
gi_291239167_Saccoglossus_kowalevskii
gi_260810520_Branchiostoma_floridae
gi_291239807_Saccoglossus_kowalevskii
gi_260793444_Branchiostoma_floridae
gi_291242484_Saccoglossus_kowalevskii
gi_291229678_Saccoglossus_kowalevskii
gi_291223815_Saccoglossus_kowalevskii
gi_196007112_Trichoplax_adhaerens
gi_72152222_Strongylocentrotus_purpuratus
gi_195998007_Trichoplax_adhaerens
gi_198419444_Ciona_intestinalis
gi_198415894_Ciona_intestinalis
gi_260786377_Branchiostoma_floridae
gi_338161004_Xenopus_laevis
gi_301609308_Xenopus_tropicalis
gi_291222148_Saccoglossus_kowalevskii
gi_291223809_Saccoglossus_kowalevskii
gi_321460550_Daphnia_pulex
gi_321478616_Daphnia_pulex
gi_241581081_Ixodes_scapularis
gi_291224467_Saccoglossus_kowalevskii
gi_321467441_Daphnia_pulex
gi_198424353_Ciona_intestinalis
gi_198419582_Ciona_intestinalis
gi_312373778_Anopheles_darlingi
gi_242023388_Pediculus_humanus_corporis
gi_115749084_Strongylocentrotus_purpuratus
gi_241672368_Ixodes_scapularis
gi_313226492_Oikopleura_dioica
gi_241998382_Ixodes_scapularis
gi_321462270_Daphnia_pulex
gi_320352592_Desulfobulbus_propionicus_DSM_2032
gi_159898230_Herpetosiphon_aurantiacus_DSM_785
gi_260426558_Citreicella_sp._SE45
gi_149919617_Plesiocystis_pacifica_SIR1
gi_114046070_Shewanella_sp._MR7
gi_148657095_Roseiflexus_sp._RS1
gi_312879450_Aminomonas_paucivorans_DSM_12260
gi_114321541_Alkalilimnicola_ehrlichii_MLHE1
gi_57642113_Thermococcus_kodakarensis_KOD1
gi_254173443_Thermococcus_sp._AM4
gi_254172615_Thermococcus_sp._AM4
gi_163848422_Chloroflexus_aurantiacus_J10fl
gi_240103523_Thermococcus_gammatolerans_EJ3
gi_309791403_Oscillochloris_trichoides_DG6
gi_309791671_Oscillochloris_trichoides_DG6
gi_309790879_Oscillochloris_trichoides_DG6
gi_159898060_Herpetosiphon_aurantiacus_DSM_785
jnetpred

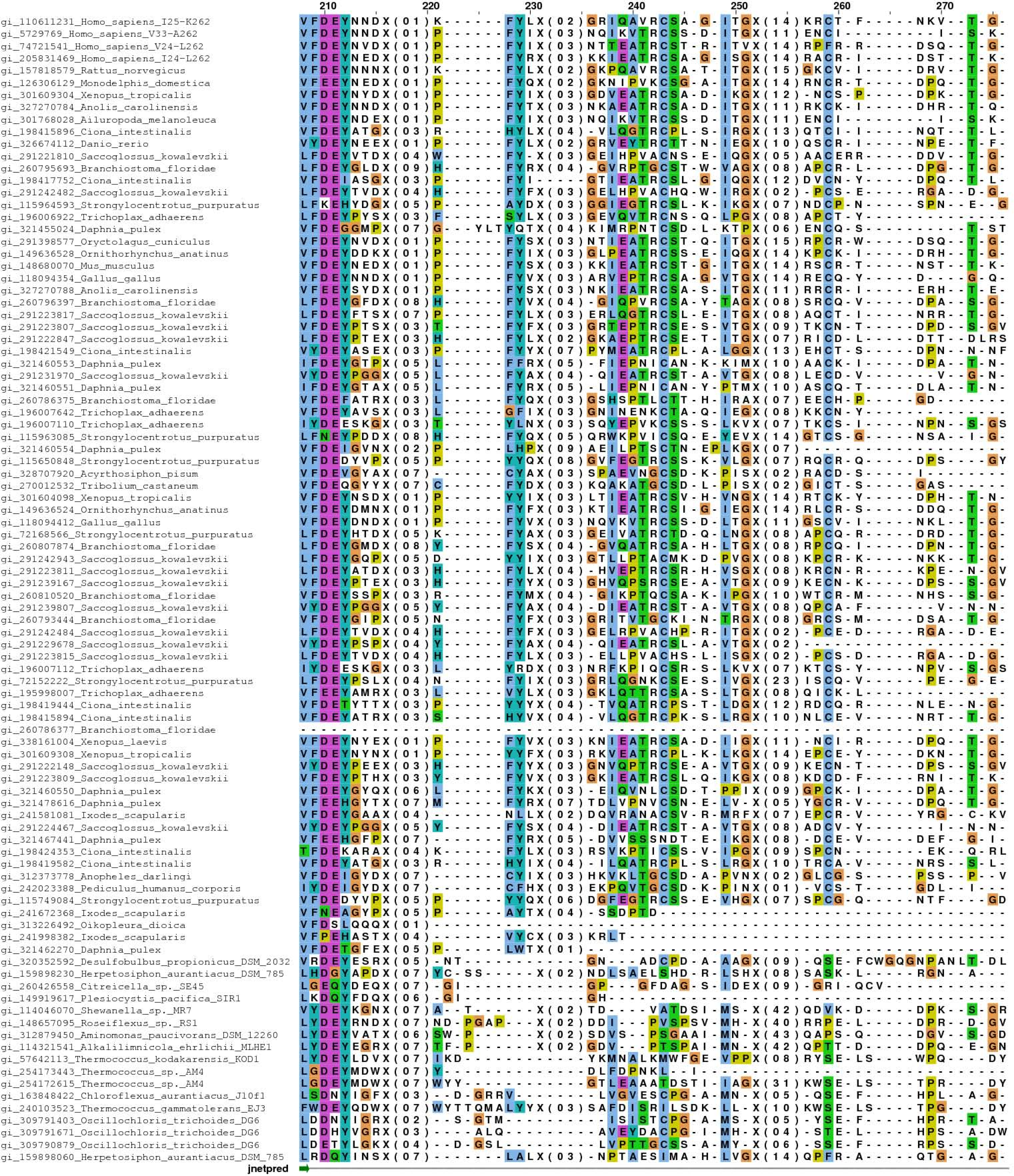
210
220
230
240
250
260
270
gi_110611231_Homo_sapiens_I25-K262
gi_5729769_Homo_sapiens_V33-A262
gi_74721541_Homo_sapiens_V24-L262
gi_205831469_Homo_sapiens_I24-L262
gi_157818579_Rattus_norvegicus
gi_126306129_Monodelphis_domestica
gi_301609304_Xenopus_tropicalis
gi_327270784_Anolis_carolinensis
gi_301768028_Ailuropoda_melanoleuca
gi_198415896_Ciona_intestinalis
gi_326674112_Danio_rerio
gi_291221810_Saccoglossus_kowalevskii
gi_260795693_Branchiostoma_floridae
gi_198417752_Ciona_intestinalis
gi_291242482_Saccoglossus_kowalevskii
gi_115964593_Strongylocentrotus_purpuratus
gi_196006922_Trichoplax_adhaerens
gi_321455024_Daphnia_pulex
gi_291398577_Oryctolagus_cuniculus
gi_149636528_Ornithorhynchus_anatinus
gi_148680070_Mus_musculus
gi_118094354_Gallus_gallus
gi_327270788_Anolis_carolinensis
gi_260796397_Branchiostoma_floridae
gi_291223817_Saccoglossus_kowalevskii
gi_291223807_Saccoglossus_kowalevskii
gi_291222847_Saccoglossus_kowalevskii
gi_198421549_Ciona_intestinalis
gi_321460553_Daphnia_pulex
gi_291231970_Saccoglossus_kowalevskii
gi_321460551_Daphnia_pulex
gi_260786375_Branchiostoma_floridae
gi_196007642_Trichoplax_adhaerens
gi_196007110_Trichoplax_adhaerens
gi_115963085_Strongylocentrotus_purpuratus
gi_321460554_Daphnia_pulex
gi_115650848_Strongylocentrotus_purpuratus
gi_328707920_Acyrthosiphon_pisum
gi_270012532_Tribolium_castaneum
gi_301604098_Xenopus_tropicalis
gi_149636524_Ornithorhynchus_anatinus
gi_118094412_Gallus_gallus
gi_72168566_Strongylocentrotus_purpuratus
gi_260807874_Branchiostoma_floridae
gi_291242943_Saccoglossus_kowalevskii
gi_291223811_Saccoglossus_kowalevskii
gi_291239167_Saccoglossus_kowalevskii
gi_260810520_Branchiostoma_floridae
gi_291239807_Saccoglossus_kowalevskii
gi_260793444_Branchiostoma_floridae
gi_291242484_Saccoglossus_kowalevskii
gi_291229678_Saccoglossus_kowalevskii
gi_291223815_Saccoglossus_kowalevskii
gi_196007112_Trichoplax_adhaerens
gi_72152222_Strongylocentrotus_purpuratus
gi_195998007_Trichoplax_adhaerens
gi_198419444_Ciona_intestinalis
gi_198415894_Ciona_intestinalis
gi_260786377_Branchiostoma_floridae
gi_338161004_Xenopus_laevis
gi_301609308_Xenopus_tropicalis
gi_291222148_Saccoglossus_kowalevskii
gi_291223809_Saccoglossus_kowalevskii
gi_321460550_Daphnia_pulex
gi_321478616_Daphnia_pulex
gi_241581081_Ixodes_scapularis
gi_291224467_Saccoglossus_kowalevskii
gi_321467441_Daphnia_pulex
gi_198424353_Ciona_intestinalis
gi_198419582_Ciona_intestinalis
gi_312373778_Anopheles_darlingi
gi_242023388_Pediculus_humanus_corporis
gi_115749084_Strongylocentrotus_purpuratus
gi_241672368_Ixodes_scapularis
gi_313226492_Oikopleura_dioica
gi_241998382_Ixodes_scapularis
gi_321462270_Daphnia_pulex
gi_320352592_Desulfobulbus_propionicus_DSM_2032
gi_159898230_Herpetosiphon_aurantiacus_DSM_785
gi_260426558_Citreicella_sp._SE45
gi_149919617_Plesiocystis_pacifica_SIR1
gi_114046070_Shewanella_sp._MR7
gi_148657095_Roseiflexus_sp._RS1
gi_312879450_Aminomonas_paucivorans_DSM_12260
gi_114321541_Alkalilimnicola_ehrlichii_MLHE1
gi_57642113_Thermococcus_kodakarensis_KOD1
gi_254173443_Thermococcus_sp._AM4
gi_254172615_Thermococcus_sp._AM4
gi_163848422_Chloroflexus_aurantiacus_J10fl
gi_240103523_Thermococcus_gammatolerans_EJ3
gi_309791403_Oscillochloris_trichoides_DG6
gi_309791671_Oscillochloris_trichoides_DG6
gi_309790879_Oscillochloris_trichoides_DG6
gi_159898060_Herpetosiphon_aurantiacus_DSM_785
jnetpred

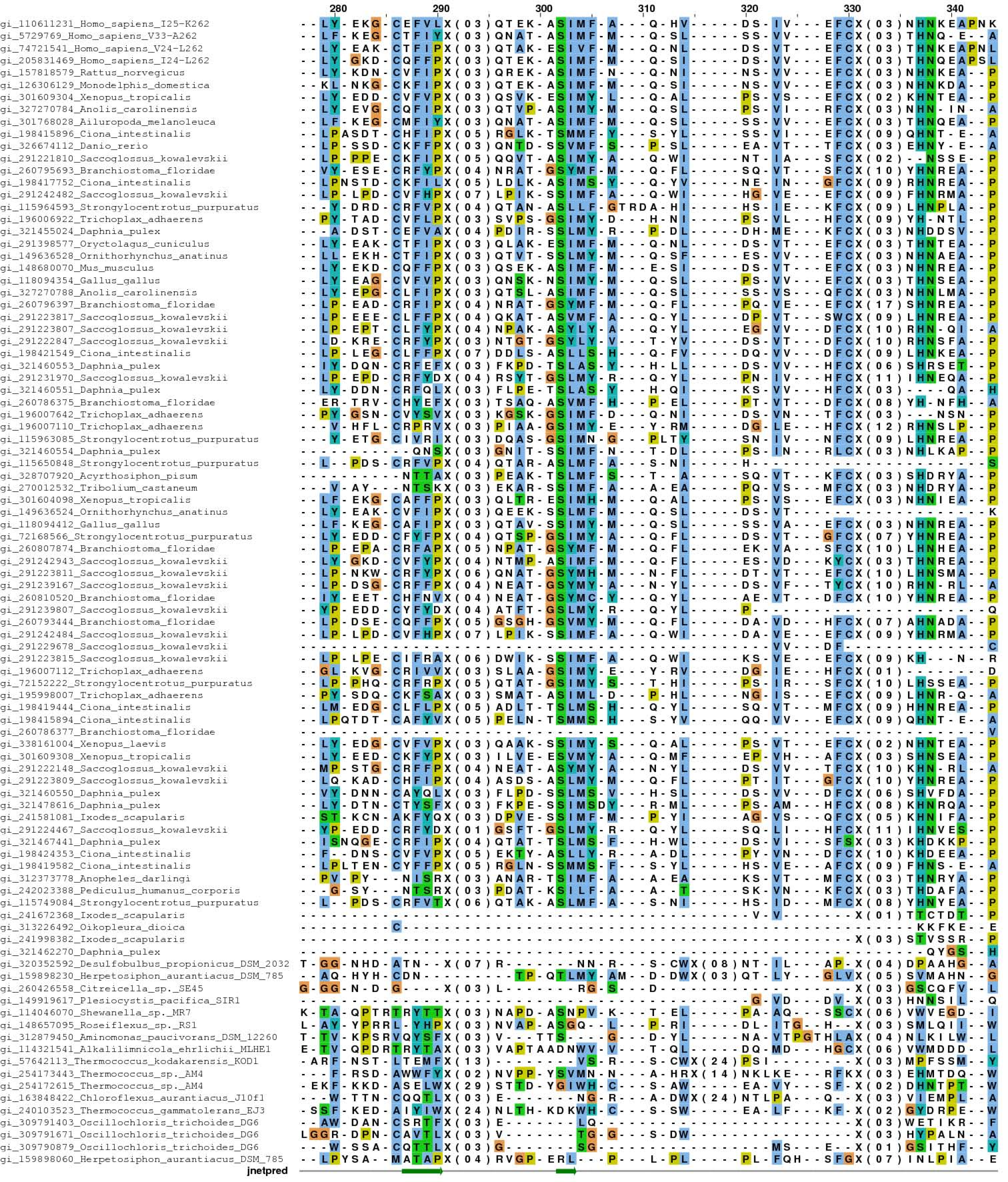

280
290
300
310
320
330
340
gi_110611231_Homo_sapiens_I25-K262
gi_5729769_Homo_sapiens_V33-A262
gi_74721541_Homo_sapiens_V24-L262
gi_205831469_Homo_sapiens_I24-L262
gi_157818579_Rattus_norvegicus
gi_126306129_Monodelphis_domestica
gi_301609304_Xenopus_tropicalis
gi_327270784_Anolis_carolinensis
gi_301768028_Ailuropoda_melanoleuca
gi_198415896_Ciona_intestinalis
gi_326674112_Danio_rerio
gi_291221810_Saccoglossus_kowalevskii
gi_260795693_Branchiostoma_floridae
gi_198417752_Ciona_intestinalis
gi_291242408_Saccoglossus_kowalevskii
gi_115964593_Strongylocentrotus_purpuratus
gi_196006922_Trichoplax_adhaerens
gi_321455024_Daphnia_pulex
gi_291398577_Oryctolagus_cuniculus
gi_149636528_Ornithorhynchus_anatinus
gi_148680070_Mus_musculus
gi_118094354_Gallus_gallus
gi_327270788_Anolis_carolinensis
gi_260796397_Branchiostoma_floridae
gi_291223817_Saccoglossus_kowalevskii
gi_291223807_Saccoglossus_kowalevskii
gi_291222847_Saccoglossus_kowalevskii
gi_198421549_Ciona_intestinalis
gi_321460553_Daphnia_pulex
gi_291231970_Saccoglossus_kowalevskii
gi_321460551_Daphnia_pulex
gi_260786375_Branchiostoma_floridae
gi_196007642_Trichoplax_adhaerens
gi_196007110_Trichoplax_adhaerens
gi_115963085_Strongylocentrotus_purpuratus
gi_321460554_Daphnia_pulex
gi_115650848_Strongylocentrotus_purpuratus
gi_328707920_Acyrthosiphon_pisum
gi_270012532_Tribolium_castaneum
gi_301604098_Xenopus_tropicalis
gi_149636524_Ornithorhynchus_anatinus
gi_118094412_Gallus_gallus
gi_72168566_Strongylocentrotus_purpuratus
gi_260807874_Branchiostoma_floridae
gi_291242943_Saccoglossus_kowalevskii
gi_291223811_Saccoglossus_kowalevskii
gi_291239167_Saccoglossus_kowalevskii
gi_260810520_Branchiostoma_floridae
gi_291239807_Saccoglossus_kowalevskii
gi_260793444_Branchiostoma_floridae
gi_291242484_Saccoglossus_kowalevskii
gi_291229678_Saccoglossus_kowalevskii
gi_291223815_Saccoglossus_kowalevskii
gi_196007112_Trichoplax_adhaerens
gi_72152222_Strongylocentrotus_purpuratus
gi_195998007_Trichoplax_adhaerens
gi_198419444_Ciona_intestinalis
gi_198415894_Ciona_intestinalis
gi_260786377_Branchiostoma_floridae
gi_338161004_Xenopus_laevis
gi_301609308_Xenopus_tropicalis
gi_291222148_Saccoglossus_kowalevskii
gi_291223809_Saccoglossus_kowalevskii
gi_321460550_Daphnia_pulex
gi_321478616_Daphnia_pulex
gi_241581081_Ixodes_scapularis
gi_291224467_Saccoglossus_kowalevskii
gi_321467441_Daphnia_pulex
gi_198424353_Ciona_intestinalis
gi_198419582_Ciona_intestinalis
gi_312373778_Anopheles_darlingi
gi_242023388_Pediculus_humanus_corporis
gi_115749084_Strongylocentrotus_purpuratus
gi_241672368_Ixodes_scapularis
gi_313226492_Oikopleura_dioica
gi_241998382_Ixodes_scapularis
gi_321462270_Daphnia_pulex
gi_320352592_Desulfobulbus_propionicus_DSM_2032
gi_159898230_Herpetosiphon_aurantiacus_DSM_785
gi_260426558_Citreicella_sp._SE45
gi_149919617_Plesiocystis_pacifica_SIR1
gi_114046070_Shewanella_sp._MR7
gi_148657095_Roseiflexus_sp._RS1
gi_312879450_Aminomonas_paucivorans_DSM_12260
gi_114321541_Alkalilimnicola_ehrlichii_MLHE1
gi_57642113_Thermococcus_kodakarensis_KOD1
gi_254173443_Thermococcus_sp._AM4
gi_254172615_Thermococcus_sp._AM4
gi_163848422_Chloroflexus_aurantiacus_J10fl
gi_240103523_Thermococcus_gammatolerans_EJ3
gi_309791403_Oscillochloris_trichoides_DG6
gi_309791671_Oscillochloris_trichoides_DG6
gi_309790879_Oscillochloris_trichoides_DG6
gi_159898060_Herpetosiphon_aurantiacus_DSM_785
jnetpred
