# Supplementary material for: CLCAs - A Family of Metalloproteases of Intriguing Phylogenetic Distribution and with Cases of Substituted Catalytic Sites
Source: PLoS One. 2013 May 9;8(5):e62272. doi: 10.1371/journal.pone.0062272 (PMC3650047; doi:10.1371/journal.pone.0062272)
Supplement: Figure S4 — Multiple sequence alignment of the full sequence set of CLCA_X domains (see Methods). Full version of lower part of Fig. 6. (PDF) [file pone.0062272.s004.pdf]

Produced with a Trial Version of PDF Annotator - www.PDFAnnotator.com

Suppl. Figure S4

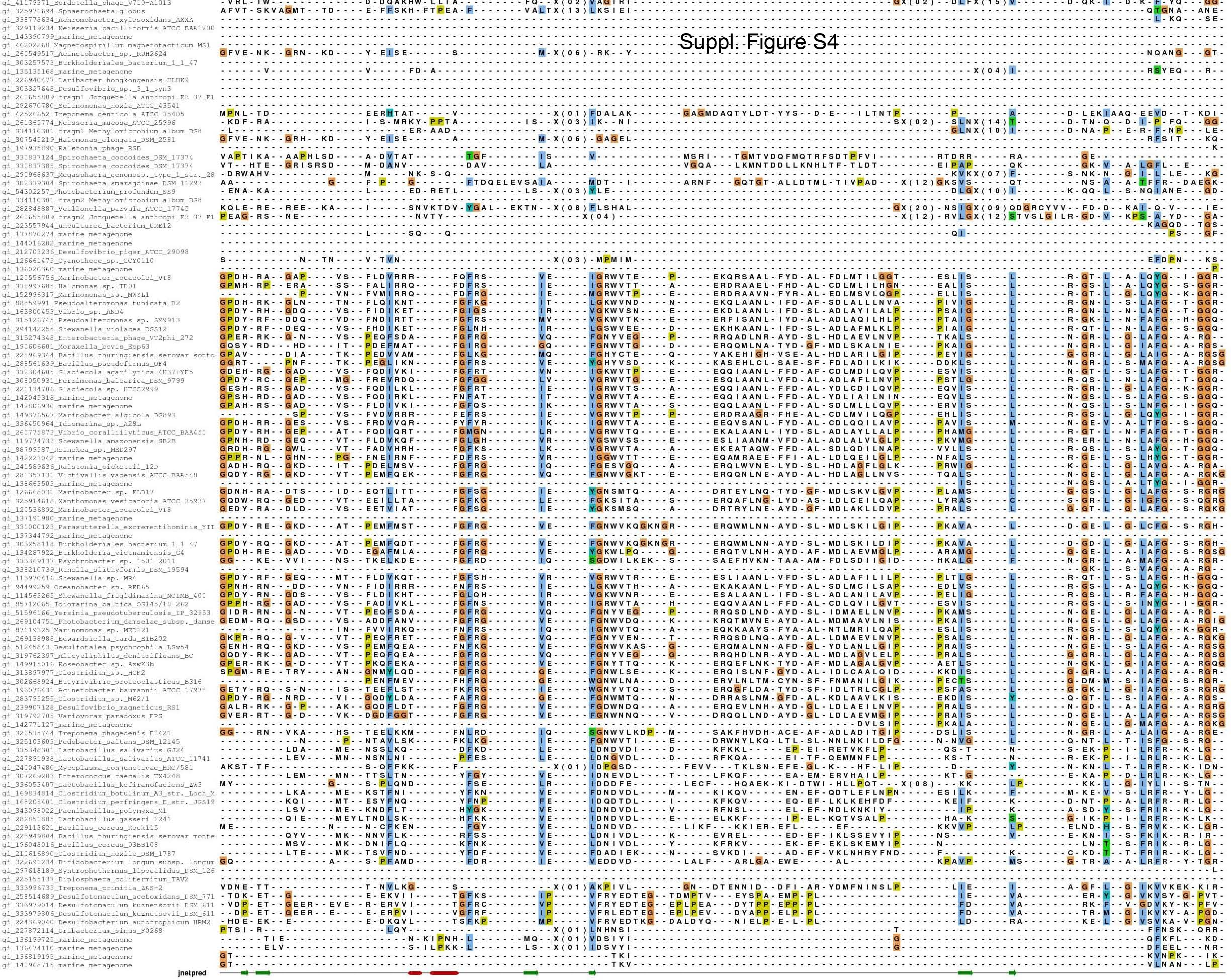

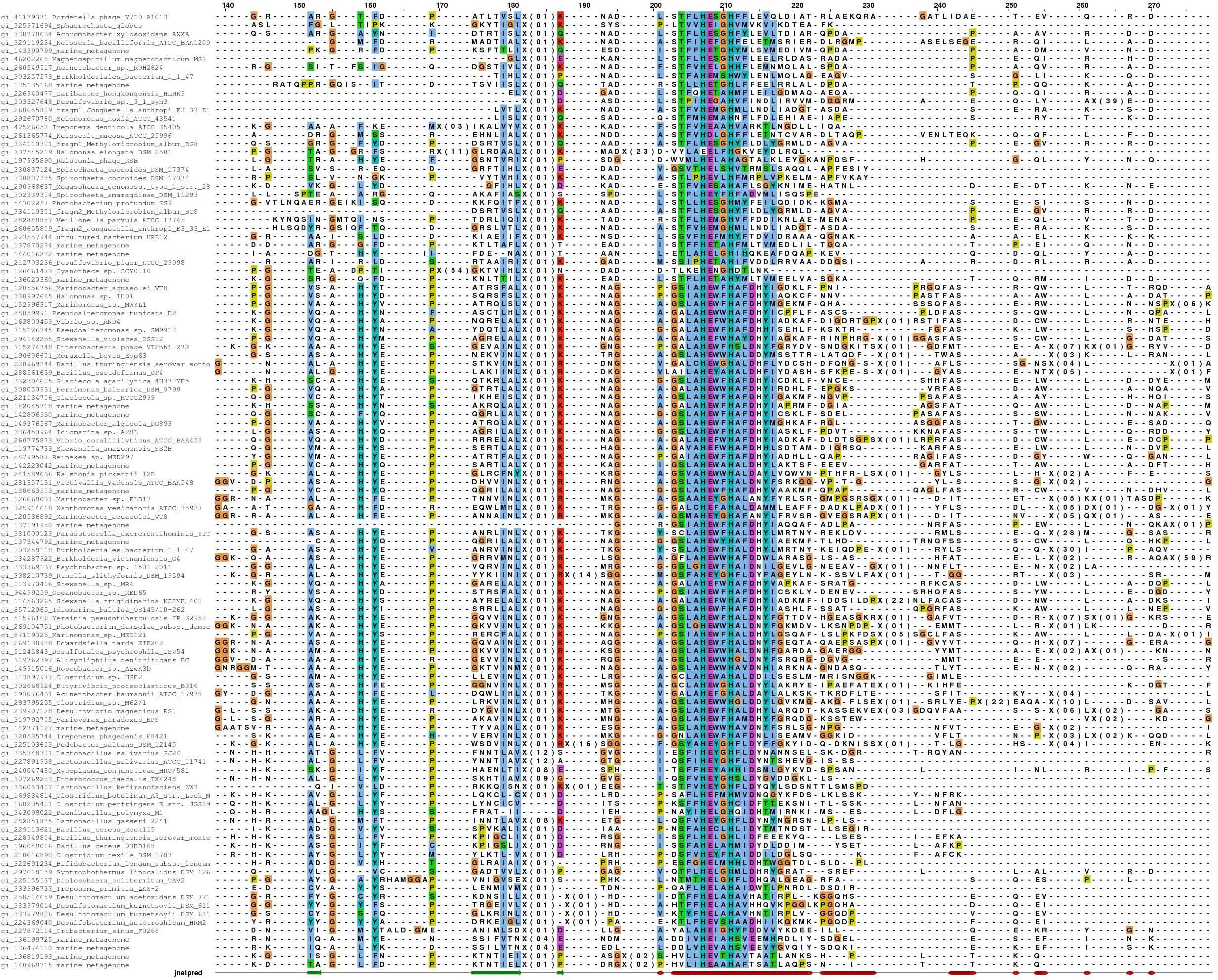
140
150
160
170
180
190
200
210
220
230
240
250
260
270
jnetpred

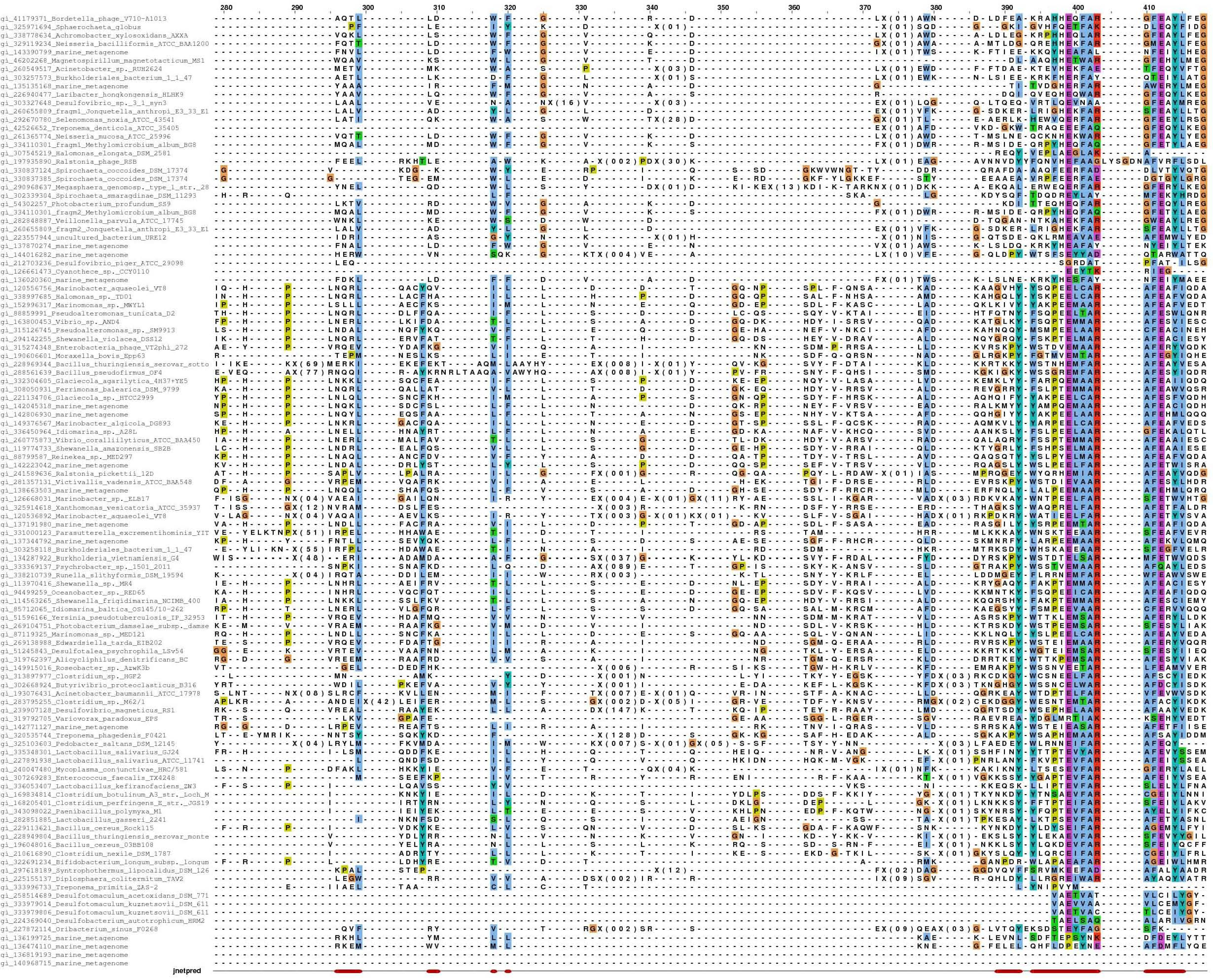
280
290
300
310
320
330
340
350
360
370
380
390
400
410
jnetpred

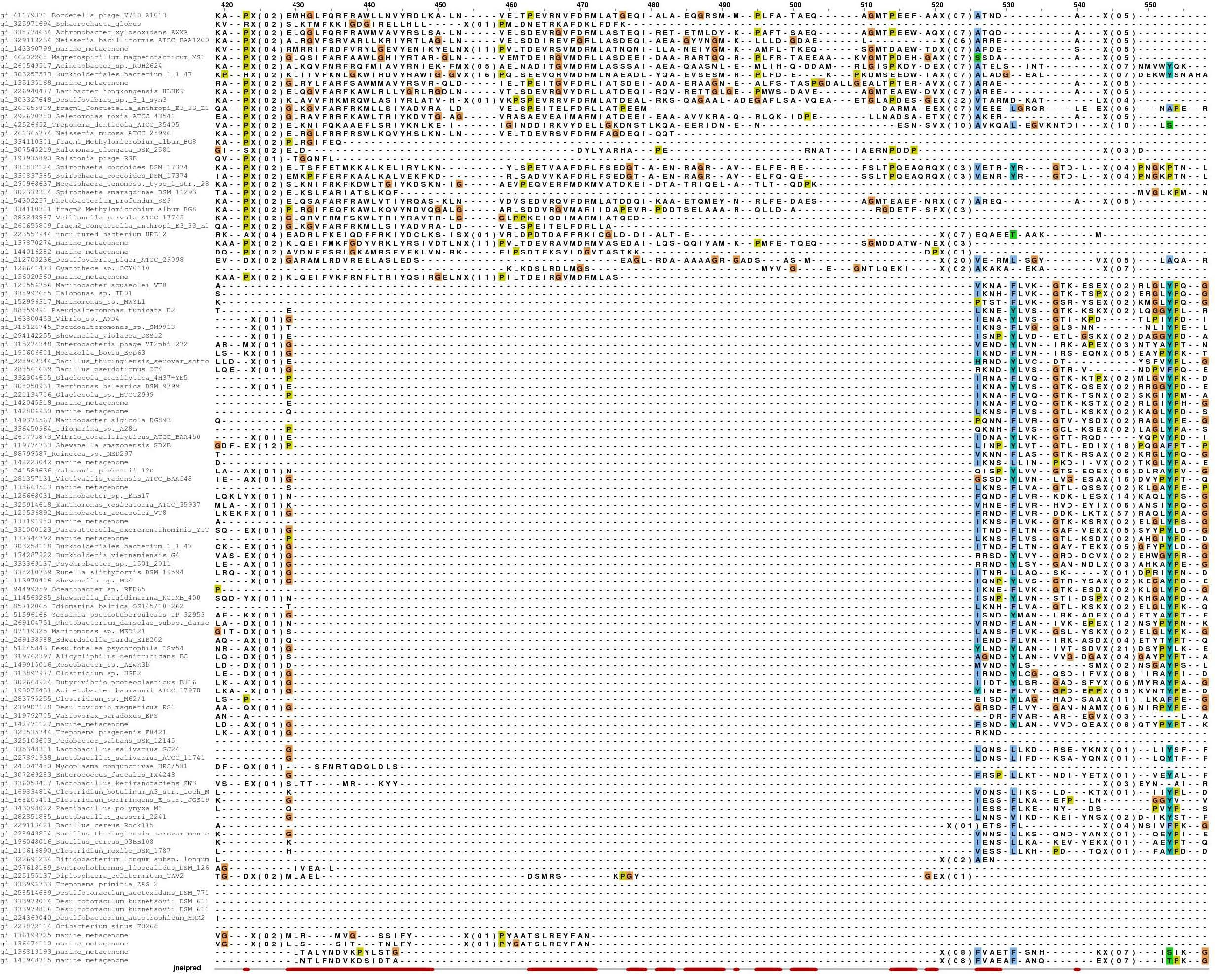
420 430 440 450 460 470 480 490 500 510 520 530 540 550
jnetpred

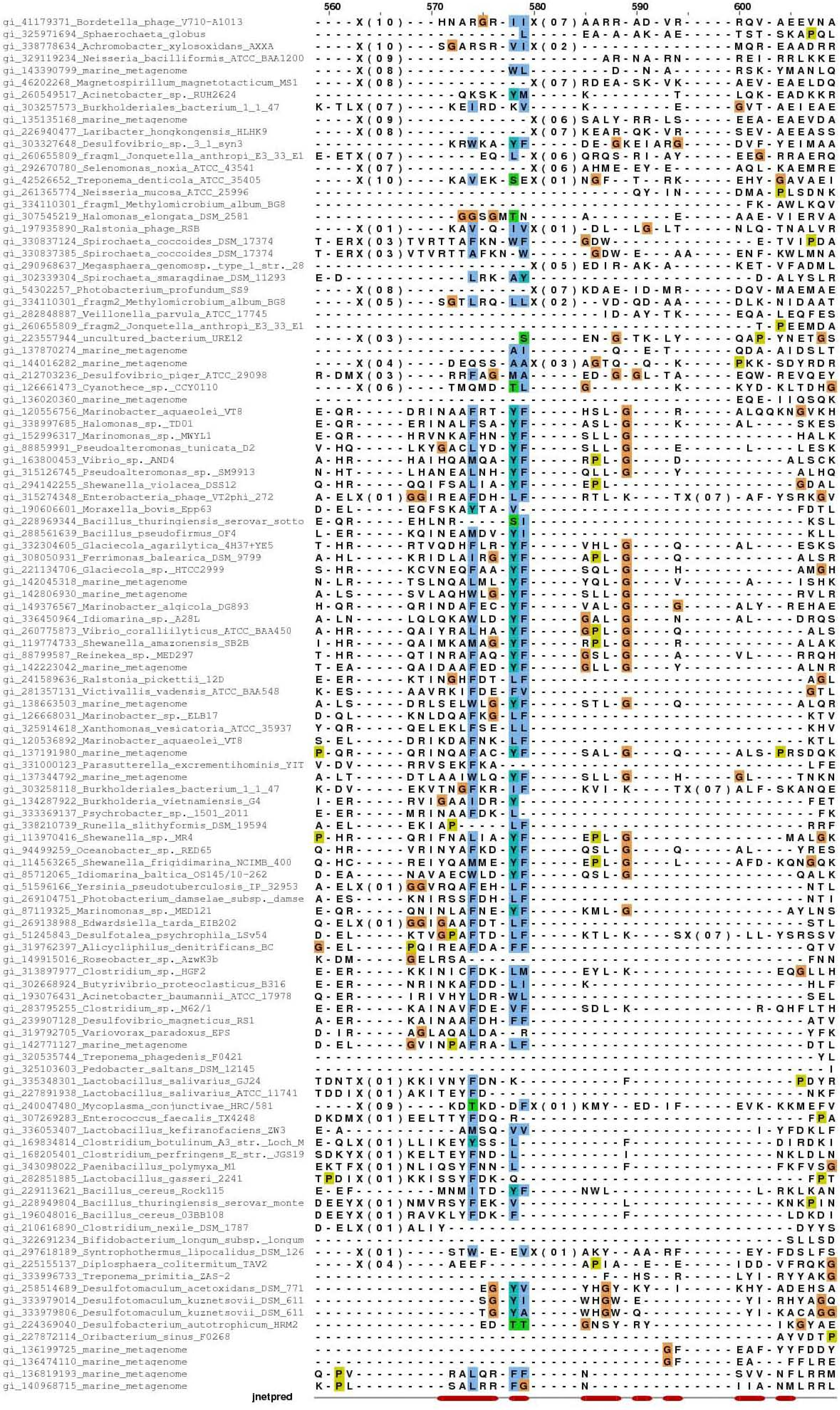

560
570
580
590
600
jnetpred
