# Supplementary material for: CLCAs - A Family of Metalloproteases of Intriguing Phylogenetic Distribution and with Cases of Substituted Catalytic Sites
Source: PLoS One. 2013 May 9;8(5):e62272. doi: 10.1371/journal.pone.0062272 (PMC3650047; doi:10.1371/journal.pone.0062272)
Supplement: Table S1 — Replacement frequencies of the critical H and E site residues in substituted HExxH motifs in the domains of the Peptidase_MA clan as defined in the Pfam database divided by corresponding replacement frequencies in proteins in general (as derived from the PAM250 substitution matrix). First column: replacement position within the HExxH motif. Values above 2 or below 0.5 in bold. (DOC) [file pone.0062272.s005.doc]

Supplementary Table S1 Replacement frequencies of the critical H and E site residues in substituted HExxH motifs in the domains of the Peptidase_MA clan as defined in the Pfam database divided by corresponding replacement frequencies in proteins in general (as derived from the PAM250 substitution matrix).

First column: replacement position within the HExxH motif. Values above 2 or below 0.5 in bold.

|  | A | C | D | E | F | G | H | I | K | L | M | N | P | Q | R | S | T | V | W | Y |
| --- | --- | --- | --- | --- | --- | --- | --- | --- | --- | --- | --- | --- | --- | --- | --- | --- | --- | --- | --- | --- |
| H1 | 0.99 | **0.25** | **0.21** | **2.21** | 0.78 | **0.42** |  | **0.36** | 0.89 | 1.44 | **0.46** | **0.40** | **0.08** | 1.20 | **2.37** | 0.93 | 1.61 | 0.56 | **0.45** | 1.36 |
| E2 | 1.98 | 0.94 | **0.21** |  | **0.38** | 0.77 | 0.55 | **0.22** | 0.87 | **2.73** | 1.04 | **0.20** | **0.12** | **3.34** | 0.5 | 1.01 | 1.13 | 0.91 | **0.20** | 0.50 |
| H5 | 0.83 | **0.29** | **0.42** | 0.93 | 1.15 | 0.73 |  | 0.62 | 1.94 | **4.89** | 0.65 | **0.35** | **0.07** | 0.69 | 1.76 | 1.38 | 0.73 | 0.62 | **0.05** | 1.43 |
